# Polyphenol-Mediated Autophagy in Cancer: Evidence of In Vitro and In Vivo Studies

**DOI:** 10.3390/ijms21186635

**Published:** 2020-09-10

**Authors:** Monica Benvenuto, Loredana Albonici, Chiara Focaccetti, Sara Ciuffa, Sara Fazi, Loredana Cifaldi, Martino Tony Miele, Fernando De Maio, Ilaria Tresoldi, Vittorio Manzari, Andrea Modesti, Laura Masuelli, Roberto Bei

**Affiliations:** 1 Saint Camillus International University of Health and Medical Sciences, Via di Sant’Alessandro 8, 00131 Rome, Italy; monica.benvenuto@unicamillus.org; 2 Department of Clinical Sciences and Translational Medicine, University of Rome “Tor Vergata”, Via Montpellier 1, 00133 Rome, Italy; albonici@med.uniroma2.it (L.A.); chiara.focaccetti@uniroma5.it (C.F.); sara.ciuffa3@gmail.com (S.C.); cifaldi@med.uniroma2.it (L.C.); demaio@med.uniroma2.it (F.D.M.); ilaria3soldi@hotmail.com (I.T.); manzari@med.uniroma2.it (V.M.); modesti@med.uniroma2.it (A.M.); 3 Department of Human Science and Promotion of the Quality of Life, San Raffaele University Rome, Via di Val Cannuta 247, 00166 Rome, Italy; 4 Department of Experimental Medicine, University of Rome “Sapienza”, Viale Regina Elena 324, 00161 Rome, Italy; sara.fazi@uniroma1.it (S.F.); laura.masuelli@uniroma1.it (L.M.); 5 Academic Department of Pediatrics (DPUO), Ospedale Pediatrico Bambino Gesù, IRCCS, Piazza Sant’Onofrio 4, 00165 Rome, Italy; 6 Department of Experimental Medicine, University of Rome “Tor Vergata”, Via Montpellier 1, 00133 Rome, Italy; miele@med.uniroma2.it

**Keywords:** polyphenols, natural compound, autophagy, cancer, cell death, cytoprotective

## Abstract

One of the hallmarks of cellular transformation is the altered mechanism of cell death. There are three main types of cell death, characterized by different morphological and biochemical features, namely apoptosis (type I), autophagic cell death (type II) and necrosis (type III). Autophagy, or self-eating, is a tightly regulated process involved in stress responses, and it is a lysosomal degradation process. The role of autophagy in cancer is controversial and has been associated with both the induction and the inhibition of tumor growth. Autophagy can exert tumor suppression through the degradation of oncogenic proteins, suppression of inflammation, chronic tissue damage and ultimately by preventing mutations and genetic instability. On the other hand, tumor cells activate autophagy for survival in cellular stress conditions. Thus, autophagy modulation could represent a promising therapeutic strategy for cancer. Several studies have shown that polyphenols, natural compounds found in foods and beverages of plant origin, can efficiently modulate autophagy in several types of cancer. In this review, we summarize the current knowledge on the effects of polyphenols on autophagy, highlighting the conceptual benefits or drawbacks and subtle cell-specific effects of polyphenols for envisioning future therapies employing polyphenols as chemoadjuvants.

## 1. Introduction

One of the hallmarks of cellular transformation is the altered mechanism of cell death. There are three main types of cell death characterized by different morphological and biochemical features, namely apoptosis (type I), autophagic cell death (type II) and necrosis (type III).

Apoptosis is an active process occurring in cells still capable of synthesizing ATP. Apoptosis is characterized by cell shrinkage, condensed chromatin, membrane blebbing and membrane phospholipids overturning, resulting in the appearance of phosphatidylserine on the outer leaflet. The DNA is cleaved by a nuclease that cuts the DNA between nucleosomes. These events are due to the action of caspase proteases that are activated during the process. The cell breaks into small membrane-bound bodies that are efficiently eliminated through phagocytosis mediated by macrophages and other cell types, before the contents of the dying cell can be released to the outside. As a result, apoptotic cell death does not usually engage an inflammatory response and is generally described as immunologically “silent” [1].

Conversely, although there may be rare forms of necrosis considered “active”, this cell death modality is generally considered passive and occurs following irreversible damage. Cell death by necrosis involves the swelling of the cell and organelles, rupture of the plasma membrane and leakage of the contents to the outside. Certain cellular components trigger inflammatory responses [1].

Autophagy, or self-eating, is an evolutionally conserved response and is a tightly regulated process involved in stress responses, such as nutritional deprivation, and removal of damaged proteins and organelles in eukaryotic cells. Autophagy is a lysosomal degradation process that differs either from the enzymatic digestion of endocytic components of extracellular derivation, or from cytoplasmic catabolic process such as proteasomal degradation [2]. The products of autophagic degradation include sugars, amino acids, fatty acids and nucleotides that, after degradation, are transported back to the cytoplasm to feed cellular metabolism and repair mechanisms [3].

Three main autophagic processes can occur depending on the way in which the components to be degraded are transferred to the lysosomes and are dependent on the type of material to be eliminated. They are macroautophagy (MA), (generally called autophagy), endosomal microautophagy/microautophagy (mA) and chaperon-mediated autophagy (CMA). In addition, MA and CMA share interactions at multiple levels and molecular machinery for the fusion of late endosomes or autophagosomes with lysosomes [4]. MA is characterized by specialized double-membrane vesicles, the autophagosomes that progressively load the material and transport it to the lysosomes by membrane fusion. Microautophagy depends on the direct uptake of cytoplasmic material through lysosomal membrane-invaginating vesicles. CMA involves the lysosomal-associated membrane protein (LAMP)-2-dependent translocation of autophagic substrates bound to cytosolic chaperones of the heat shock protein (HSP) family across the lysosomal membrane [5]. The autophagic process consists of five steps that include induction (i), nucleation (ii), vesicle lengthening and maturation (iii), vesicle fusion (iv) and, finally, degradation and recycling (v).

**Figure 1:**
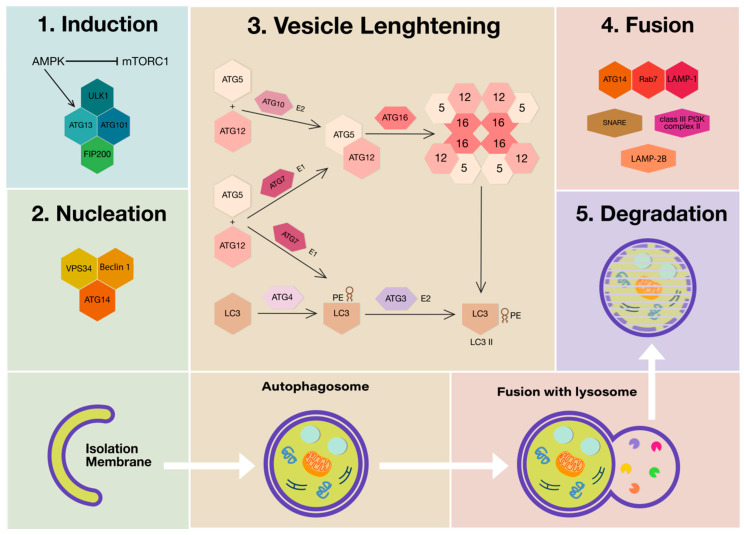
Schematic representation of mammalian autophagy molecular mechanisms. The inhibition of the mammalian target of rapamycin complex 1 (mTORC1) by AMP-activated protein kinase (AMPK), following nutrient deprivation due to accumulation of AMP, allows autophagy to start (1. Induction). AMPK sequentially phosphorylates and activates autophagy-activating kinase 1 (ULK1), autophagy-related (ATG)13, ATG101, Vps34 and Beclin 1 to nucleate the formation of phagophore (2. Nucleation). The recruitment of two ubiquitin-like conjugation systems, ATG10 (E2) and ATG7 (E1), catalyzes the formation of the multimeric complex (ATG12, ATG5, and ATG16). This complex, together with microtubule-associated protein 1A/1B-light chain 3 (LC3)-phosphatidylethanolamine (PE), derived from the action of ubiquitin-like conjugation systems ATG7 (E1), are part of the vesicle elongation process (3. Vesicle lengthening). Next, mammalian ATG8 family members LC3 and GABARAPs mediate the phagophore membrane closure (4. Fusion). Autophagosomes fuse with lysosomes under the regulation of cytoskeleton elements. Various proteins including ATG14, lysosomal-associated membrane protein (LAMP)-1, LAMP-2B, Rab7 and soluble N-ethylmaleimide-sensitive factor-activating membrane fusion protein (SNARE) participate to the formation of the autolysosome, where the degradation of cargo occurs by the action of lysosomal enzymes (5. Degradation). Abbreviations: AMPK, AMP-activated protein kinase; ULK1, unc-51-like autophagy-activating kinase 1; Vps34, phosphatidylinositol 3-kinase catalytic subunit type 3 (PIK3C3/Vps34); ATG, autophagy-related protein; ATG16L1, autophagy-related 16-like 1; PE, phosphatidylethanolamine; MAP1LC3B/LC3B, microtubule-associated protein 1 light chain 3 beta; GABARAPs, γ-aminobutyric acid type A receptor- associated proteins; LAMP-1, lysosomal-associated membrane protein 1; Rab7, Ras-related protein 7; SNARE, soluble N-ethylmaleimide-sensitive factor-activating membrane fusion protein.

This process is tightly regulated by the recruitment of autophagy-related (ATG) proteins and is normally repressed by the mechanistic target of rapamycin complex 1 (mTORC1). Conversely, in response to decreased ATP levels and consequent AMP accumulation, mTORC1 is inhibited by AMP-activated protein kinase (AMPK). Therefore, the triggering of the autophagic process implies the inhibition of mTORC1 and consequent derepression and phosphorylation of components such as unc-51-like autophagy-activating kinase 1 (ULK1), ATG13 and ATG101, which play an essential role in the induction step of autophagy [6,7]. In addition to ULK1 phosphorylation, AMPK phosphorylates phosphatidylinositol 3-kinase catalytic subunit type 3 (PIK3C3/ Vps34) and Beclin 1 (BECN1), which promote the elongation and forming of the phagophore membrane following synthesis of phosphatidylinositol 3-phosphate [8,9]. Then, the formation of the autophagosome involves both the covalent linkage of ATG5, ATG12 and autophagy-related 16-like 1 (ATG16L1) and the conjugation of phosphatidylethanolamine (PE) to microtubule-associated protein 1 light chain 3 beta (MAP1LC3B/LC3B) [10]. LC3 is a member of ATG8 family proteins, it is synthesized as pro-LC3 and cleaved by ATG4 to generate a diffuse cytosolic form known as LC3 I, then LC3 I is activated by ATG7 and transferred to ATG3 [11]. The complex ATG5-ATG12 can conjugate LC3 into a membrane-bound PE group, giving rise to LC3 PE, known as LC3 II, which has been used widely as autophagosome marker [12]. Although the complete mechanism of phagophore membrane closure is relatively unknown, LC3 and γ-aminobutyric acid type A receptor-associated proteins (GABARAPs), of mammalian ATG8 family members, are required to form the autophagosome [13]. Autophagosomes fuse with lysosomes under the regulation of cytoskeleton elements. In this step, different proteins such as class III phosphatidylinositol 3-kinase (PI3K) complex II, ATG14, LAMP-1, LAMP-2B, Ras-related protein 7 (Rab7), and soluble N-ethylmaleimide-sensitive factor-activating membrane fusion protein (SNARE) participate in the formation of the autolysosome, where the degradation of cargo occurs by the action of lysosomal enzymes [14,15] (Figure 1).

Microautophagy needs chaperon proteins and the cargo is directly internalized into small vesicles that originate from the surface of the lysosome. Two pathways are known, the first (defined as endosomal microautophagy) requires the endosomal sorting complexes for transport (ESCRT), but, instead, the second (defined as microautophagy) is independent from ESCRT. Microautophagy also requires some components of MA machinery for cargo targeting and internalization, including ATG7, ATG8 and ATG9 [16]. Moreover, endosomal microautophagy differs from CMA for its independence from the LAMP-2-specific splicing variant.

CMA is characterized by the direct delivery of cytosolic proteins that are targeted for degradation to the lysosome. These substrates reach the lysosomal lumen through a protein translocation complex at the lysosomal membrane [17]. Substrates can cross the lysosomal membrane due to a dedicated molecular machinery that involves a specific splicing isoform of LAMP-2, namely LAMP-2A [18]. In fact, substrates bind LAMP-2A monomers on the cytosolic side of the lysosome and stimulate the formation of an oligomeric LAMP-2A translocation complex [19]. The LAMP-2A complexes are then stabilized by a lysosomal pool of HSP90 and the lysosomal HSPA8 acts as an acceptor for CMA substrates in preventing their retrotranslocation to the cytosol [20]. CMA can degrade only soluble proteins bearing a KFERQ-like motif bound to HSPA8 [21], but not macromolecules such lipids, nucleic acids, or proteins integral to membranes or organelles [22]. Therefore, CMA is a HSPA8-dependent autophagic mechanism that relies mainly on LAMP2A-mediated cargo translocation across the lysosomal membrane, although other forms of microautophagy can be LAMP-2A-independent [23].

Autophagy is also a process in which potentially harmful cytoplasmic or disposable entities undergo lysosomal degradation, allowing cells to eliminate both permeabilized mitochondria and byproducts of normal cellular process in order to preserve physiological homeostasis [24]. Despite the fact that autophagy is categorized as type II cell death, paradoxically, autophagy can denote a cell survival mechanism in stressful conditions. Indeed, in mammalian cells, autophagic responses can often mediate robust cytoprotective effects [25]; therefore, in several cases, the disruption of autophagy machinery can accelerate cell death.

A novel form of autophagy-dependent non-apoptotic cell death was recently identified and termed autosis. Although a specific marker for autosis has not been identified, this form of autophagy is characterized by distinctive morphological features. Among them, the main feature is the separation of the inner and outer nuclear membranes with the enlargement of the perinuclear space. Other features encompass enhanced cell–substrate adherence, dependence on Na+/K+-ATPase, and occurrence in very specific conditions (starvation, autophagy-inducing peptide treatment, cerebral hypoxia ischemia in vivo) [26,27].

The role of autophagy in cancer is controversial and has been associated with both the induction and the inhibition of tumor growth. Autophagy can exert tumor suppression through the degradation of oncogenic proteins, suppression of inflammation and chronic tissue damage and ultimately by preventing mutations and genetic instability [12,25]. On the other hand, tumor cells require autophagy for survival in cellular stress conditions. Indeed, autophagy-deficient tumor cells show a pronounced survival disadvantage in response to metabolic stress in comparison to autophagy-proficient tumor cells [28]. In addition, activation of oncogenic pathways induces an increase in cell energy consumption by promoting autophagy in transformed cells and thus ensuring their survival.

Since baseline autophagy at low rate of degradation guarantees cell protection against stressful stimuli, pharmacological agents or dietary interventions that inhibit or activate autophagy are receiving great interest as new therapies for different pathological conditions, including malignant, cardiovascular, autoimmune and neurodegenerative diseases [29,30].

In this review, we summarize the current knowledge on the effects of polyphenols on autophagy, highlighting the conceptual benefits or drawbacks and subtle cell-specific effects of polyphenols for envisioning future therapies employing polyphenols as chemoadjuvants.

## 2. Polyphenols

Polyphenols are natural compounds found in foods and beverages of plant origin, including fruits, vegetables, spices, cereals, nuts, legumes, olives, tea, coffee, and wine [31,32].

**Figure 2:**
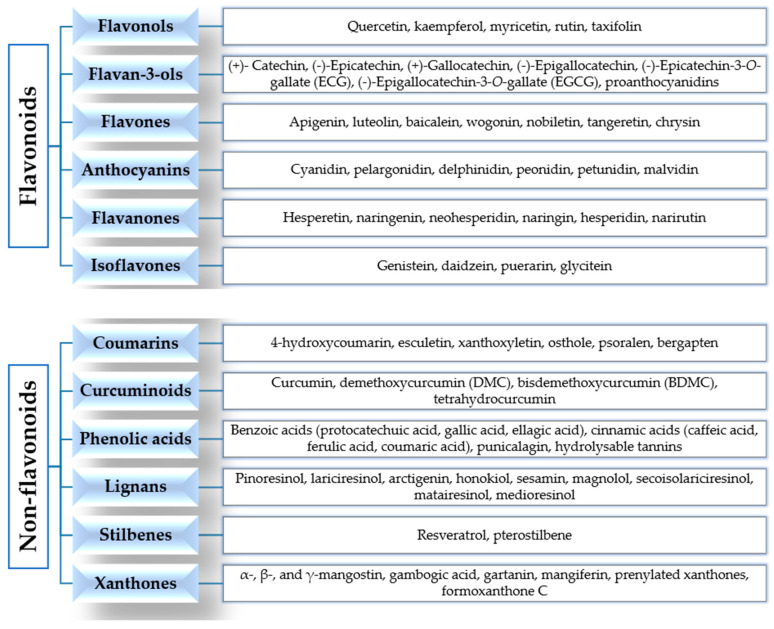
Classification of polyphenols.

According to their chemical structures, polyphenols can be classified into flavonoids and non-flavonoids (Figure 2).

Flavonoids are widely present in our diet and are formed from phenylalanine [33,34]. Their chemical structure consists of 15 carbon atoms with aromatic rings A and B connected by a three-carbon bridge, forming a heterocyclic ring (ring C) [35]. They are divided into subclasses, according to the different functional groups, the level of oxidation of ring C and the different connections between rings B and C [34,36]. The main subclasses are flavonols, flavan-3-ols, flavones, anthocyanins, flavanones and isoflavones [34,36].

Flavonols are the most abundant flavonoids in our diet, mainly present as glycosylated forms. The main members of this subclass, found in fruits, edible plants, tea and wine, are quercetin, kaempferol and myricetin [31,34,36]. Flavan-3-ols are a chemically complex subclass, which comprises monomeric, oligomeric, and polymeric compounds, including (+)-catechin, (−)-epicatechin, (+)-gallocatechin, (−)-epigallocatechin, (−)-epicatechin-3-*O*-gallate (ECG), (−)-epigallocatechin-3-*O*-gallate (EGCG), and proanthocyanidins. They mainly occur in fruits, berries, nuts, cereals, chocolate, tea and red wine [34,36,37]. Flavones are mainly present as 7-*O*-glycosides in foods, including parsley, celery, onion, garlic, chamomile, tea and citrus fruits. Examples of this subclass are apigenin, luteolin, baicalein, wogonin, nobiletin, tangeretin and chrysin [34,37]. Anthocyanins are more than 550 compounds in nature, including cyanidin, pelargonidin, delphinidin, peonidin, petunidin and malvidin. Berries, cherries, red grapes, currants and red wine are the main food sources [34,38,39]. The flavanones found in citrus fruits (oranges, grapefruits, lemons, mandarins) are aglycone compounds (hesperetin and naringenin), neohesperidosides (neohesperidin and naringin) and rutinosides (hesperidin and narirutin) [34,37,40]. Isoflavones have a chemical structure similar to estrogens and they are mainly present in soybeans, soy products and leguminous plants. The main members are genistein, daidzein and glycitein [34,41,42].

The class of non-flavonoids comprises coumarins, curcuminoids, phenolic acids, lignans, stilbenes and xanthones. Coumarin (C_9_H_6_O_2_, 2H-1-benzopyran-2-one) and its derivatives are alpha-benzopyrones, which can be divided into: simple coumarins, furanocoumarins, pyranocoumarins, and dicoumarins. They are found in the fruits, leaves, flowers, stems and roots of several plants [43,44]. Curcumin (1,7-bis-(4-hydroxy-3-methoxyphenyl)-1,6-heptadiene-3,5-dione) is the main member of the curcuminoids subclass, derived from the rhizome of the plant *Curcuma longa* and found in the spice turmeric. Curcumin is a pleiotropic molecule and it is a “multifunctional drug”, because it is able to modulate multiple targets and signaling pathways involved in cancer [34,45,46]. Phenolic acids are divided into hydroxycinnamic acids (caffeic acid, ferulic acid, *p*-coumaric acid and sinapic acid), present in coffee, fruits and cereal grains, and hydroxybenzoic acids (protocatechuic acid and gallic acid), found in a few edible plants. Gallic acid is the biosynthetic precursor of hydrolysable tannins (gallotannins and ellagitannins), mainly found in mangoes and red fruits [34,37]. Lignans are phytoestrogens, because of their structural similarities with estrogens. They are mainly present in flaxseed, sesame seed, linseed, cereals, vegetables, fruits, red wines, tea, coffee and olive oil. Examples include pinoresinol, lariciresinol, arctigenin, sesamin, magnolol, honokiol, secoisolariciresinol, matairesinol, medioresinol [34,47,48,49]. Stilbenes are phytoalexins, with limited occurrence in our diet. The main member is resveratrol (3,5,4′-trihydroxystilbene), found in grapes, berries, plums, peanuts and pine nuts, and has many biological properties [34,50,51].

Xanthones (9H-xanthen-9-one or dibenzo-γ-pirone) are secondary metabolites of plants of the families *Gentianaceae*, *Guttiferae*, *Moraceae*, *Clusiaceae* and *Polygalaceae*. They comprise simple xanthones, xanthone glycosides, prenylated xanthones, xanthonolignoids, bisxanthones, and miscellaneous xanthones. Examples include mangiferin (1,3,6,7-tetrahydroxyxanthone-C2-β-D-glucoside), α-, β-, γ-mangostin, gambogic acid and gartanin [52,53].

Many studies have positively associated the consumption of dietary polyphenols with the prevention of many diseases, including cancer [32]. Indeed, polyphenols possess many biological activities that are important for human health, including antimicrobial, antioxidant, anti-inflammatory, antiviral, anticancer, and immunomodulatory functions [34,36,54,55,56,57].

## 3. In Vitro- and In Vivo-Mediated Autophagy by Polyphenols

**Figure 3:**
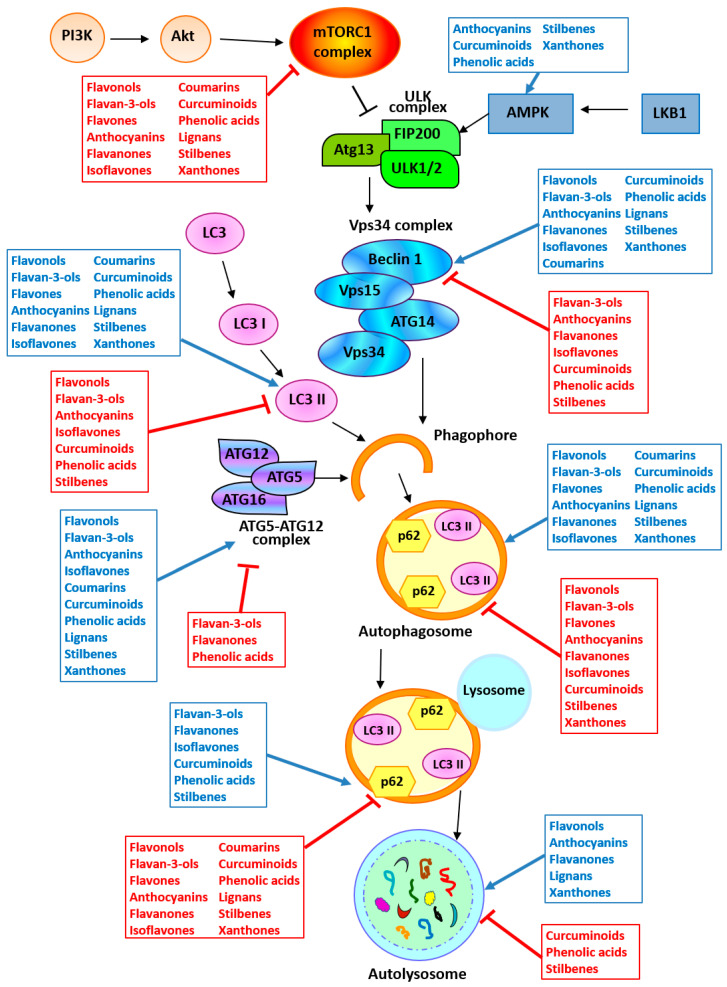
Targeting autophagy by polyphenols. The arrow and the inhibition arc indicate a positive and a negative activity of the polyphenols reported in the boxes, respectively. Abbreviations: AMPK, AMP-activated protein kinase; ATG, autophagy-related protein; Fip200, Fak family kinase interacting protein of 200 kD; LC3, Microtubule-Associated Protein 1 Light Chain 3; LKB1, serine/threonine liver kinase B1 (STK11); mTORC1, mammalian target of rapamycin complex 1; PI3K, phosphatidylinositol 3-kinase; ULK, unc-51-like autophagy-activating kinase; Vps, vacuolar protein sorting-associated protein.

In vitro and in vivo studies underlying the potential of polyphenols in modulating autophagy in cancer are summarized in Table 1 and Figure 3.

### 3.1. Flavonoids

#### 3.1.1. Flavonols

Several studies investigated the biological effects of the flavonol quercetin on autophagy in various types of cancer. Quercetin is the main member of the flavonoids subclass of flavonols and it is the most common flavonol in the diet of the Western population [327]. Klappan et al. reported that quercetin (90 µM) induced autophagy-mediated cell death through the inhibition of the proteasome activity and of the mTOR signaling pathway in epithelial cancer cells (MCF-7, HeLa) [58]. Quercetin was able to induce a strong cytotoxic effect in Burkitt’s lymphoma. It inhibited the PI3K/Akt/mTOR pathway and decreased *c*-Myc expression, leading to apoptosis of Burkitt’s lymphoma cells. In addition, quercetin (100 µM) induced a complete autophagic flux, which contributed to the partial degradation of *c*-Myc and thus to its reduced expression [59]. The effect of quercetin on tumor metastasis through autophagy has been investigated in breast cancer. Quercetin (30 µM) promoted autophagy by the inactivation of the Akt/mTOR pathway in MCF-7 and MDA-MB-231 cells. In addition, the in vivo administration of quercetin (50 mg/kg, intraperitoneally (i.p.), twice daily for a month) was able to reduce the size of the tumors and to reduce the level of the protein Beclin 1 and of phospho-Akt/Akt ratio in tumor tissues in a breast cancer xenograft mouse model [60]. Moon et al. demonstrated that quercetin enhanced lung cancer cell death induced by tumor necrosis factor-related apoptosis-inducing ligand (TRAIL) through the autophagic flux activation in A549 cells. Quercetin treatment (20–80 µM) induced the formation of autophagosomes and an increase in LC3 II expression levels [61]. The in vivo anticancer efficacy of quercetin was investigated in human leukemia by Calgarotto et al. The authors demonstrated that quercetin (120 mg/kg, i.p., every 4 days, for 21 days) and green tea (100 mg/kg, by gavage, daily, for 21 days) reduced tumor growth by the activation of apoptosis and autophagy in human leukemia (HL-60) xenografts from Non-Obese Diabetic/severe combined immunodeficiency disease (NOD/SCID) mice [62]. Another study reported that quercetin (30–50 µM) was able to act in synergy with the drug sorafenib in inducing apoptosis and autophagy in human anaplastic astrocytoma (MOGGCCM) and glioblastoma multiforme (T98G) cell lines, thus suggesting the potential use of this dual therapy for the treatment of these tumors [63].

Different studies provided evidence that quercetin induced the activation of protective autophagy, since the inhibition of autophagy led to the enhancement of apoptotic cell death induced by the flavonol [64,65,66,67,68,69,70,71,328,329]. Kim et al. reported that quercetin (25–100 µM) promoted the activation of the intrinsic pathway of apoptosis, through the activation of c-Jun N-terminal kinase (JNK) in human malignant glioma U373MG cells. Additional experiments showed that pretreatment with the autophagy inhibitor chloroquine increased apoptotic cell death, thus suggesting that quercetin promoted protective autophagy [64]. The same findings were reported in human malignant glioma U87 and U251 cells and also in an in vivo xenograft model (intracranial injection of rat glioma cells C6 in rats and treatment with quercetin at 100 mg/kg, i.v., daily) by Bi et al. [65]. Similarly, Wang et al. reported that quercetin induced the activation of apoptosis and protective autophagy in gastric cancer cells by inactivating the Akt/mTOR pathway and HIF-1α signaling. Treatment of AGS (10–40 µM) and MKN28 (40–160 µM) cells with quercetin induced the activation of apoptosis and the formation of double membrane autophagic vacuoles, the conversion of LC3 I to LC3 II and the accumulation of LC3 II. The in vivo experiments confirmed these findings, as shown by the increase in the expression and accumulation of LC3 II in gastric tumor xenografts from mice treated with quercetin (50 mg/kg, i.p., daily, for 24 days). However, the authors also reported that the administration of the autophagic inhibitor chloroquine or the selective ablation of ATG5 or Beclin 1 using small interfering RNA (siRNA) increased quercetin-induced apoptotic cell death, suggesting that autophagy played a protective role against quercetin-induced apoptosis [66]. Treatment with quercetin (50 µM) induced a pro-survival autophagy and apoptosis in primary effusion lymphoma cells (PEL), and increased the cytotoxic effect of the proteasomal inhibitor bortezomib [67]. Quercetin promoted protective autophagy in ovarian cancer cells. Quercetin (40–80 µM) was able to induce endoplasmic reticulum stress-mediating mitochondrial apoptosis and protective autophagy through the phospho-signal transducer and activator of transcription (STAT)3/Bcl-2 axis in CAOV3 cells and primary ovarian cell P#1. In addition, the authors demonstrated that the autophagy inhibitor 3-Methyladenine (3-MA) potentiated the anti-cancer effects of quercetin (80 mg/kg, i.p., twice a week, for 4 weeks) in ovarian cancer mice xenografts [68]. Similarly, it was found that quercetin induced apoptosis and cytoprotective autophagy in P39 leukemia cells (50 µM), in HL-60 acute myeloid leukemia (AML) cells (100 µM) and in HeLa cells (50 µM). In fact, the use of the autophagy inhibitor, 3-MA, significantly enhanced quercetin-mediated apoptotic cell death in these cell lines [69,70,71]. The use of quercetin in combination with resveratrol has been evaluated in human hepatoblastoma HepG2 cells. Quercetin (100 µM) induced a potent activation of autophagy, which was attenuated by increasing the dose of resveratrol (1–100 µM), when used in combination. The authors suggested that the attenuation of quercetin-induced autophagy by resveratrol could led to an enhancement of apoptosis [329]. Taylor et al. demonstrated that quercetin (25 µM), in combination with sodium butyrate (1 mM), was able to enhance the apoptotic cell death through the blockade of the protective autophagy under nutrient starvation in rat C6 and human T98G glioblastoma cells [328].

All these studies suggest that the inhibition of autophagy may be a novel strategy to enhance the anticancer activity of quercetin in various types of tumors.

The effects on autophagy of quercetin derivatives were evaluated in different studies. Enayat et al. reported that a novel semi-synthetic derivative of quercetin, with improved bioavailability and solubility, 3,7-dihydroxy-2-[4-(2-chloro-1,4-naphthoquinone-3-yloxy)-3-hydroxyphenyl]-5-hydroxychromen-4-one (CHNQ), was able to induce cancer cell death in colorectal cancer HCT-116 and HT-29 cell lines. CHNQ was threefold more cytotoxic than quercetin and it activated apoptosis and reactive oxygen species (ROS)-induced autophagy (25 µM and 40 µM, for HCT-116 and HT-29, respectively). In particular, the authors observed a complete autophagy in HCT-116 cells and an incomplete autophagy in HT-29 cells with the successful lipidation of LC3 II but impaired acidic vesicular organelle (AVO) formation [72]. It has been reported that quercetin can be converted in a novel quercetin derivative, 8-C-(E-phenylethenyl)quercetin (8-CEPQ), in onion/beef soup. It was reported that this derivative (15 µM) was able to induce autophagic cell death through the activation of the extracellular signal-regulated kinase (ERK) pathway in human colon cancer cell lines (SW620 and HCT-116) [73]. The effect of a novel *O*-alkylated derivative of quercetin, 7-*O*-geranylquercetin (GQ), has been investigated in non-small-cell lung cancer (NSCLC) A549 and NCI-H1975 cell lines. GQ (25–35 µM) activated apoptosis and autophagy through the generation of ROS in these cell lines. GQ was able to induce autophagosomes formation, to promote the expression of LC3 II and Beclin 1, and to inhibit the expression of p62. In addition, the autophagy induced by GQ contributed to apoptosis activation, because the treatment with chloroquine or Beclin 1 siRNA inhibited GQ-induced apoptosis [74].

Several studies explored the effects of quercetin-3-*O*-β-D-galactopyranoside, or hyperoside, a flavonol glycoside mainly found in plants of the genera *Hypericum* and *Crataegus* [75,76,330]. Fu et al. showed that hyperoside (0.5–2 mM) induced autophagy and apoptosis in human NSCLC cells. In particular, hyperoside increased the levels of LC3 II and autophagosome numbers and decreased the levels of p62. In addition, hyperoside-induced autophagy was associated with the inhibition of the Akt/mTOR/p70S6K signaling pathway and the activation of the ERK1/2 signaling pathways. It was also reported that hyperoside-induced apoptosis of A549 cells was at least partly dependent on autophagy [75]. Similarly, Zhu et al. investigated the effect of this flavonol in ovarian cancer cells. Hyperoside was able to induce autophagy-associated cell death in ovarian cancer cells. The authors showed that hyperoside (50–100 µM) induced progesterone receptor membrane component (PGRMC)1-dependent autophagy in SKOV-3 and HO-8910 cells. In addition, autophagy induced by the flavonol is essential for the activation of apoptosis in these cell lines [76]. Conversely, another study reported that hyperoside (50 µM for 48 h) was able to induce apoptosis but not autophagy in pancreatic cancer cells (MIA PaCa-2 cells) [330].

Isorhamnetin (ISO), an immediate 3′-*O*-methylated metabolite of quercetin in mammals, is found in plants of the *Polygonaceae* family and exhibits anti-tumor effects. It has been reported that ISO was able to induce autophagy and mitochondria-dependent apoptosis in human NSCLC A549 cells. Treatment with ISO (2–8 µM) increased the levels of LC3 II, Beclin 1 and the number of autophagosomes in a dose-dependent manner. However, the use of autophagy inhibitors demonstrated that ISO induced a pro-survival type of autophagy. The pre-treatment of lung cancer cells with autophagy inhibitors (3-MA and chloroquine) suppressed autophagy and enhanced ISO-induced cancer cell apoptosis. In addition, the in vivo anti-tumor activity of ISO (0.5 mg/kg/day; i.p.) was evaluated in a xenograft mouse model in the presence or absence of autophagy inhibitors, thus confirming that inhibition of autophagy enhanced the growth inhibitory effect of ISO in this type of cancer [77].

Rutin, quercetin-3-*O*-rutinoside or vitamin P, is a flavonol abundant in edible plants, such as onion, orange, lemon, apple and green tea. Zhang et al. demonstrated that rutin (50–200 µM) increased temozolomide (TMZ) cytotoxicity through the inhibition of JNK-mediated protective autophagy induced by TMZ in human glioblastoma multiforme cell line D54MG. Rutin (20 mg/kg, i.p., for 18 days) also increased TMZ-mediated cytotoxicity in vivo in subcutaneous and intracranial mouse tumor models. Thus, rutin could be used in combination with TMZ for the treatment of glioblastoma multiforme [78].

Another study explored the effects of taxifolin or dihydroquercetin, a dihydroflavonol, in combination with the anticancer agent andrographolide in HeLa cells. They reported that taxifolin (100 µM) increased the caspase-dependent apoptosis induced by andrographolide. In addition, taxifolin inhibited the ROS-dependent protective autophagy activated by the anticancer agent, thus leading to the improvement of its cytotoxic effects [79].

Recent studies showed that the delivery of quercetin by nanoparticles enhanced its efficacy with reduced side effects. The effects of gold–quercetin into poly (DL-lactide-*co*-glycolide) nanoparticles were explored in cancer cells. In one study, quercetin nanoparticles treatment suppressed the in vitro (30–40 µg/mL) and in vivo (40–80 mg/kg, i.p., daily) growth of human neuroglioma U87 cells, by inducing cell autophagy and apoptosis through the inhibition of Akt/mTOR signaling pathway [80]. Luo et al. investigated the in vitro and in vivo effects of quercetin nanoparticles in cervical cancer cells. They showed that quercetin nanoparticle (10–20 µg/mL) treatment inhibited cervical cancer Caski cell growth by the induction of autophagy, as indicated by the formation of autophagosomes, and apoptosis. In addition, quercetin nanoparticles inhibited the growth of cervical cancer cells in a xenograft mouse model [81].

Other studies evaluated the effect of the flavonol kaempferol, found in several plant derivatives (e.g., apples, onion, leeks, citrus, grapes, gingko biloba, St. John’s wort, red wine), on several types of cancer [82,83,84,85,86]. The antitumor effects of kaempferol were evaluated in hepatocellular carcinoma (HCC) cells. This polyphenol inhibited SK-Hep-1 cell proliferation and induced the activation of autophagic cell death, but not apoptosis. Indeed, kaempferol (50–100 µM) induced the formation of double membrane vacuoles, lysosomal compartments, AVOs, and increased the protein expression levels of LC3 II, ATG5, ATG7, ATG12 and Beclin 1. The kaempferol-induced autophagy was associated with the upregulation of phospho-AMPKα and the downregulation of phospho-Akt and phospho-mTOR protein levels [82]. Han et al. demonstrated that kaempferol was able to suppress the proliferation of human NSCLC cells (A549), by promoting apoptosis and autophagy. The flavonol (20–50 µM) induced the increased expression of cleaved caspases, Bax/Bcl-2, ATG7, LC3 II/I, Beclin 1 and the decrease in the protein p62. In addition, the authors demonstrated that the kaempferol-induced autophagy promoted cell apoptosis, because apoptotic cells were eliminated by the treatment with the autophagy inhibitor 3-MA. Kaempferol affected cell growth, apoptosis and autophagy through the increase in miR-340, which led to the inhibition of the PI3K/Akt pathway [83]. Similarly, Zhang and colleagues reported that kaempferol inhibited the growth of gastric cancer SNU-216 cell line, with the activation of autophagy, but not apoptosis. The results showed the decrease in the protein expression levels of p62 and the increase in ATG7, LC3 II/I, and Beclin 1 after treatment with kaempferol (50 µM). The growth inhibitory effect and the activation of autophagy were achieved by the inhibition of MAPK/ERK and PI3K pathways and by the increase in the expression of miR-181 in this type of cancer as well [84]. Kaempferol (50 µM) was also able to promote autophagic cell death, as shown by the increase in the conversion of LC3 I to LC3 II, and by the decrease in p62, in AGS and SNU-638 gastric cancer cells. The promotion of the autophagic cell death by kaempferol was achieved by the activation of the IRE1–JNK–DNA damage-inducible transcript 3 protein (CHOP) signaling pathway and by the inhibition of G9a [85]. A recent study reported that kaempferol and caffeic acid phenethyl ester (CAPE), from propolis, inhibited cell growth and induced apoptosis and autophagy in RKO and HCT-116 colon cancer cell lines [86].

Juglanin, a flavonol extracted from the crude Polygonum aviculare, has been investigated for its effects on breast cancer by Sun et al. They demonstrated that juglanin (2.5–10 µM) inhibited cell growth through the stimulation of apoptosis and autophagy, as shown by the activation of caspases, by the formation of autophagosomes and by the increase in LC3 II. In addition, the authors reported that the activation of the two types of cell death was mediated by the ROS/JNK signaling pathway in human breast cancer cells (MCF-7, SK-BR-3 cells). Finally, the in vivo effects of juglanin were investigated. The results showed that juglanin (5 and 10 mg/kg, i.p., for 7 days) inhibited the growth of human breast cancer xenografts, with an increase in the levels of activated caspases, LC3 I and II, and JNK phosphorylation [87].

Park et al. investigated the effect of a flavonol extracted from *Broussonetia papyrifera*, Kazinol A, on bladder cancer. Kazinol A exerted cytotoxic effects in T24 and cisplatin-resistant T24R2 human bladder cancer cells through the induction of apoptosis and autophagy. Indeed, it has been reported that the flavonol (20 µM) modulated AMPK/mTOR pathways (increased AMPK phosphorylation and decreased mTOR phosphorylation) and led to autophagic cell death, as shown by the formation of autophagosomes and the conversion of LC3 I to LC3 II [88].

Two studies demonstrated the activation of autophagic cell death in cutaneous squamous cell carcinoma A431 cells (25–100 µM) and HCC (10–50 µM) by dihydromyricetin (DHM), a natural flavonoid from *Ampelopsis grossedentata*. The results showed the activation of the autophagic flux and the upregulation of LC3 II and Beclin 1 [89,90]. Conversely, it has been reported that DHM (100 μM) induced cytoprotective autophagy in SK-MEL-28 human melanoma cells by activating the NF-κB pathway, and that the pharmacological inhibition of DHM-induced autophagy sensitized SK-MEL-28 cells to DHM-induced apoptotic cell death [91]. The same results were obtained by Fan et al. in their study on head and neck squamous cell carcinoma (HNSCC) cells, showing that DHM (50 μM) induced apoptotic cell death and autophagy and that the inhibition of autophagy led HNSCC cells to DHM-induced apoptotic cell death [92].

#### 3.1.2. Flavan-3-ols

Several studies reported the induction of autophagy as a mechanism of cell death by flavan-3-ols in different cancer cells. For example, the effects of *Hibiscus sabdariffa* leaf polyphenolic (HLP) extract, which mainly contain ECG were evaluated in melanoma cells. The results of the study showed that HLP (100–250 μg/mL) and ECG (100 µM) induced the activation of intrinsic and extrinsic pathways of apoptosis, as well as autophagic cell death in A375 cells and thus led to the inhibition of cell proliferation [93]. It has been reported that EGCG (20 µM) inhibited cell proliferation of SSC-4 human oral squamous cell carcinoma (OSCC), and induced cell death with the activation of apoptosis and autophagy [94]. It was also shown that EGCG (10–20 µM) affected breast cancer 4T1 cell growth in vitro and in vivo by promoting apoptosis and autophagy, and by inhibiting enzymes involved in the glycolytic pathway [95]. High levels of alpha-fetoprotein (AFP) are indicators of poor prognosis for HCC. Zhao et al. demonstrated that EGCG (25–50 µM) was able to induce cytoplasmic AFP aggregation, to inhibit AFP secretion, and also to activate autophagy, which promoted the degradation of AFP aggregates in HCC HepG2 cells [96]. The combined effect of radiation and EGCG was explored recently. It was found that EGCG (12.5 µM) improved the sensitivity of HCT-116 colorectal cancer cells to radiation, by inducing autophagy and Nrf2 nuclear translocation [97]. The effect of the combined treatment with EGCG (20 µM), a low strength pulsed electric field (PEF) and a low energy ultrasound (US) has been evaluated in the human HCC cell line HepG2 and in the human pancreatic cancer cell line PANC-1. The triple treatment was able to cause the cell death by activating apoptosis and autophagy [98]. Recently, Xie et al. synthesized a novel ECG analog, 4-(S)-(2,4,6-trimethylthiobenzyl)-EGCG (JP8), capable of inducing cell death in B16-F10 melanoma cells. JP8 (20 µM) induced cell death through the activation of autophagy, mediated by intracellular ROS accumulation. In addition, JP8 (25 and 50 mg/kg, i.p., daily, for 21 days) suppressed tumor growth in a C57BL/6 mouse melanoma model [101]. Grube et al. reported that EGCG at 500 µM (6–12 h) induced a strong activation of autophagy and apoptosis in primary glioblastoma cells (GBM15, GBM16), whereas had no effect at the central nervous system (CNS)-achievable concentrations (100 nM for 6 days). The authors hypothesized that catechins might have short-term effects at CNS-achievable concentrations, acting as mild stressors. Indeed, the results showed that catechins (100 nM) induced initial autolysosome formation within the first 6 h of incubation, which decreased during the following 6 h. Thus, the regular consumption of green tea probably led to activation of protective pathways that confer stress resistance [99].

In fact, other studies reported the induction of cytoprotective autophagy by flavan-3-ols. Green tea extract (GTE), which contains some flavan-3-ols, such as (−)-epigallocatechin, EGCG, (−)-epicatechin, and ECG, induced protective autophagy in NSCLC A549 cells. The induction of the protective autophagy led to insensitivity of A549 cells to GTE treatment, even at high doses (150 µM). Indeed, the blockade of autophagy with bafilomycin A in combination with GTE led to increased necrotic cell death [102]. Another study reported that Polyphenon E^®^, a standardized GTE, led to a transient induction of autophagy within 12 h after treatment (dose of 35 µg/mL), as a survival response to overcome endoplasmic reticulum stress in prostate cancer PNT1a cells; then, cells were committed to anoikis [103]. Moreover, Satoh et al. demonstrated that EGCG (40–500 µM) induced the apoptotic cell death of human malignant mesothelioma cells (EHMES-10, EHMES-1, ACC-meso, Y-meso and MSTO-211H) through the production of ROS. However, they also showed that EGCG induced a cytoprotective autophagy. EGCG activated the autophagic flux, but the inhibition of this pathway by chloroquine led to an enhancement of EGCG-induced cell death [100].

The use of tea polyphenols in combination with anticancer drugs has also been investigated. The results of the studies showed the increase in the cytotoxicity induced by the anticancer drugs through two different modalities of action of flavan-3-ols on autophagy. Indeed, flavan-3-ols inhibited the drug-induced cytoprotective autophagy or enhanced the drug-induced autophagy. Gu et al. demonstrated that the treatment with tea polyphenols could be used in combination with epirubicin to improve the efficacy of this therapy in bladder cancer. It was reported that tea polyphenols (100 µM) inhibited epirubicin-induced autophagy and sensitized T24 cells and BIU87 cells to epirubicin-induced apoptosis [104]. Wang et al. reported the same findings in human castration-resistant prostate cancer PC-3 and DU145 cell lines. Pretreatment of cells with tea polyphenols (20 μM) inhibited docetaxel-induced cytoprotective autophagy, through the activation of the mTOR pathway, and improved the efficacy of the therapy with docetaxel [105]. Similarly, it was found that EGCG was able to increase the anticancer effect of doxorubicin, by inhibiting the doxorubicin-induced autophagy in hepatoma Hep3B cells (treatment with 10–40 µg/mL of EGCG) and in a subcutaneous Hep3B cells xenograft tumor model (daily intragastric treatment with 50 mg/kg of EGCG) [106]. Recently, Wang et al. demonstrated that EGCG (20 µg/mL) increased the efficacy of doxorubicin in osteosarcoma (SaoS2 and U2OS cells), by reducing the pro-survival autophagy induced by the drug, through the downregulation of the SOX2OT variant 7 [107]. Meng et al. also demonstrated that EGCG overcame resistance to gefitinib in NSCLC. EGCG (34 µM) increased A549 cell death by inhibiting both gefitinib-induced autophagy and ERK phosphorylation [108].

Other studies demonstrated that EGCG increased the cytotoxicity of cisplatin and oxaliplatin, by enhancing autophagy. In particular, it was reported that EGCG (100 µM) improved the autophagic cell death induced by the two drugs in DLD-1 and HT-29 human colorectal cancer cells, as indicated by the formation of autophagosomes and by the increase in LC3 protein levels and AVOs [109]. EGCG (50 µM) also induced apoptosis and autophagy in cisplatin-resistant oral cancer CAR cells, by suppressing the Akt/STAT3 pathway and multidrug resistance 1 (MDR1) signaling [110].

However, other studies reported that EGCG antagonized the cytotoxic effects of other novel anticancer treatments, by inducing autophagy. For example, the effects of the combined treatment with the proteasome inhibitor bortezomib and the polyphenol EGCG was explored by Modernelli et al. They found that EGCG (5 or 50 µM) antagonized the cytotoxic effect of bortezomib on prostate cancer PC-3 cells, by increasing the activation of autophagy. This led to the protection of cells from apoptosis by the mitigation of endoplasmic reticulum stress and to the reduction in the upregulation of CCAAT/enhancer binding protein homologous protein (DNA damage-inducible transcript 3 protein (CHOP)), an endoplasmic reticulum stress marker [111]. It has also been demonstrated that the anti-cancer therapeutic agent TRAIL can induce apoptotic cancer cell death by the activation of death receptors. A study reported that EGCG (5–20 µM) was able to protect human colorectal HCT-116 cancer cells from the TRAIL-induced apoptosis, by downregulating death receptors, through the activation of autophagic flux. The result was confirmed by the pharmacological inhibition of autophagy with chloroquine, that led to the sensitization of cancer cells to TRAIL-induced cell death upon EGCG treatment. The authors suggested further consideration of the use of EGCG, as an autophagy activator, when used in combination with TRAIL-based anticancer therapy [112].

#### 3.1.3. Flavones

Several studies evaluated the role of flavones on autophagy. Brunelli et al. investigated the effects of increasing concentrations of 8-prenylapigenin and its 3′-methoxylated analogue isocannflavin B (IsoB) on the proliferation of estrogens sensitive ER^+^ T47-D and insensitive ER^−^ MDA-MB-231 cells. They showed that IsoB (25 µM) induced autophagic cell death in ER^+^ breast cancer cells [113]. Apigenin (20–80 μM) restored autophagy in primary human epidermal keratinocytes (HEKs) and cutaneous squamous cell carcinoma cell line COLO16 exposed to UVB radiation, thus suggesting a photoprotective role of this flavone on UVB-induced skin cancer [114]. Ruela de Sousa et al. showed the antitumor activity of apigenin (100 μM) in erythroid subtype TF1 leukemia cells by initiating autophagy but not apoptosis [115]. Our group investigated the role of apigenin in malignant mesothelioma (MM-F1, MM-B1 and H-Meso-1) cell lines, showing that this compound (50 μM) induced apoptosis, but not autophagy in these cell lines. Indeed, the expression levels of Beclin 1 and p62 remained unchanged upon apigenin treatment [116]. Xiaping et al. demonstrated the development of autophagosomes in apigenin treated cisplatin-resistant colon cancer HT-29 cells (15–60 µM), indicating that this flavonoid induced autophagic process, as corroborated by the upregulation of the autophagy-related proteins Beclin 1 and LC3 II and the suppression of p62 expression [117]. The same results were obtained in human papillary thyroid carcinoma BCPAP cells in which apigenin (12.5–50 µM) led to a markedly increase in LC3 II, Beclin 1 accumulation and p62 degradation [118]. In addition, it was demonstrated that apigenin (10–40 µM) induced autophagy and apoptosis through inhibition of the PI3K/Akt/mTOR pathway in HCC cells. However, the use of the 3-MA autophagy inhibitor enhanced the apigenin-induced apoptosis, revealing the protective effect of autophagy against cell death [119]. Lee et al. reported the same effect of apigenin (6.25–50 µM) in colon cancer HCT-116 cells [120]. The inhibition of mTOR/p70S6k pathway, for the activation of autophagy was also exerted by wogonin (50 µM), a flavone from *Scutellaria baicalensis,* in human nasopharyngeal carcinoma cells (NPC-TW076 and NPC-TW039). In these cells, the activation of autophagy by wogonin had interference with the apoptotic death, induced through the inhibition of Akt/cRaf/ERK pathway [121]. In a similar way, baicalein, another flavone found in the root of *Scutellaria baicalensis* (100 and 200 µM), induced apoptosis via endoplasmic reticulum stress and triggered cytoprotective autophagy in HCC SMMC-7721 and Bel-7402 cells [124]. The protective role of baicalein-induced autophagy in preventing cell death was also reported in HCC HepG2 cells and in ovarian HEY and A2780 cancer cells. In particular, baicalein (12.5–50 µM) triggered autophagy, by inhibiting the Akt/mTOR pathway [125,126]. Li et al. demonstrated that baicalein (25–100 µM) induced cytoprotective autophagy also in OSCC Cal27 cells. Indeed, the use of inhibitors of autophagy enhanced baicalein-induced apoptosis [127]. In addition, Chen et al. demonstrated that the hydroxylated polymethoxyflavone 5-demethylnobiletin (5-DMN; 12.5 μM), found in citrus plants, activated cytoprotective autophagy through the JNK pathway in CL1-5 and NSCLC A549 cells. JNK activation disrupted the Bcl-2-Beclin 1 association, releasing Beclin 1 and activating autophagy. Pretreatment with 3-MA potentiated 5-DMN-induced apoptosis [133]. Moreover, luteolin induced apoptotic cell death and the autophagic process in MET4 cells (50 µM). However, the use of the autophagy inhibitor chloroquine resulted in a significant increase in luteolin-induced apoptosis, thus suggesting a cytoprotective role for autophagy [134]. Rafatian et al. evaluated the effect of salvigenin on oxidative stress-mediated apoptosis and autophagy in human neuroblastoma SH-SY5Y cells. The results showed that salvigenin (25–50 µM) inhibited H_2_O_2_-induced apoptosis and enhanced autophagy, in order to help cells to survive cellular stress. Thus, salvigenin-induced autophagy played a role as a pro-survival mechanism [139].

Conversely, several studies demonstrated the induction of flavone-mediated autophagy as a cell death mechanism in several types of cancer. Liu et al. reported that baicalein (10–80 µM) inhibited proliferation of glioma U251 cells, by inducing autophagy and apoptosis through the activation of the AMPK pathway [128]. Baicalein (10–80 µM) also suppressed the growth of undifferentiated thyroid cancer cells by inducing apoptosis and autophagy [129]. Similarly, the 7-*O*-glucuronide of baicalein (40–160 µM), triggered both apoptosis and autophagy to promote cell death of human HCC SMMC-7721 cells [140]. Aryal et al. demonstrated that baicalein (5 µg/mL) induced cell death, mainly by autophagy, in human cancer cells PC-3, MDA-MB-231 and DU145, as shown by the formation of autophagosomes and the activation of autophagic flux. Moreover, baicalein activated AMPKα leading to ULK1 activation and downregulating both protein and mRNA levels of mTOR and Raptor [130]. Yan et al. observed that baicalein (10, 20, 40 µM) induced the formation of autophagic vacuoles and increased the levels of LC3 II and Beclin 1, through the inhibition of the PI3K/Akt pathway, in MCF-7 and MDA-MB-231 breast cancer cells. These results were also confirmed in vivo in breast cancer xenograft mice (100 mg/kg baicalein, orally, once daily for 21 days) [131]. Two other flavones induced cell death by autophagy in breast cancer cells as well [142,143]. Lewinska et al. studied the antitumor activity of diosmin, a citrus fruit flavonoid, in MCF-7, MDA-MB-231 and SK-BR-3 breast cancer cells. They demonstrated that the treatment with diosmin induced oxidative stress and DNA damage leading to cytostatic (5 and 10 μM) and cytotoxic (20 μM) autophagy [142]. Moreover, it has been demonstrated that seed extracts from *Euterpe oleracea* Mart., a plant from the Amazon region, promoted autophagy (10, 20 and 40 μg/mL) in the MCF-7 breast cancer cell line, indicating the antitumorigenic potential of this compound [143]. The blocking of the Akt signaling pathway by baicalin (100–200 µM) to activate autophagic cell death was also demonstrated in human bladder cancer T24 cells. Baicalin downregulated the phospho-Akt (Ser473) protein level and Akt kinase activity and increased the ATG complex, LC3 and Beclin 1 expression [141]. The induction of autophagic cell death by inhibiting the Akt/mTOR/p70S6K pathway was also reported for delicaflavone, a biflavonoid from *Selaginella doederleinii*, in A549 and PC-9 lung cancer cells. In particular, treatment with 40 μg/mL delicaflavone increased autophagosome numbers, the LC3 II/LC3 I ratio and downregulated the expression of phospho-Akt, phospho-mTOR, and phospho-p70S6K [144]. Luteoloside, a naturally flavonoid isolated from the medicinal plant *Gentiana macrophylla*, also induced autophagic cell death (60 µM) in A549 and H292 NSCLC cells by inhibiting the Akt/mTOR/p70S6K signaling pathway and this resulted in the overexpression of Beclin 1 and LC3 II and in a reduced expression of p62 [145]. Glychionide-A, another flavone extracted from several plant species, inhibited the growth of PANC-1 pancreatic cancer cells (7–28 µM), by promoting both apoptosis and autophagy [146]. Similarly, other studies showed the capacity of isoorientin (20–80 μM), a C-glycosyl flavone, and glycosylflavonoid isovitexin (12.5–50 μg/mL) to inducing apoptosis and autophagy, leading to cell death of HepG2 and SK-Hep1 HCC cells [147,148].

Moreover, several studies reported that luteolin induced cell death through the activation of autophagy. For example, Park et al. reported that luteolin induced endoplasmic reticulum stress-mediated apoptosis and Beclin 1-independent autophagy in NCI-H460 lung carcinoma cells [135]. Luteolin (20 µM) induced autophagic flux in human liver cancer cells Huh7 by upregulating LC3 II and inhibiting p62 expression, thus sensitizing cells to TRAIL-induced cell death [136]. The same effects of luteolin were obtained by Cao et al. in human liver cancer SMMC-7721 cells. The treatment (25–100 µM) increased the number of apoptotic cells and intracellular autophagosomes and increased the expression of LC3 II and Beclin 1. Co-treatment with the autophagy inhibitor chloroquine reduced the effects of luteolin on cell apoptosis [137]. It has been demonstrated that wogonin (4–16 µM) exerted its anticancer effects on human colorectal cancer cells (SW48) by inducing both autophagic and apoptotic processes, as shown by the formation of autophagosomes, the increase in Beclin 1 and LC3 II expression [122]. Wogonin (10, 50 and 200 µM) also improved the oxaliplatin-induced cell death through the enhancement of autophagy in BGC-823 human gastric cancer cells [123]. Another flavone from *Scutellaria baicalensis* Georgi, wogonoside (250 µM), induced cell death in human glioblastoma cells (U251MG and U87MG) by promoting apoptosis and by enhancing autophagic flux. The activation of autophagy was shown to be required for the wogonoside-induced apoptosis and it was mediated through the activation of p38 MAPK, inhibition of PI3K/Akt/mTOR/p70S6K pathways and by ROS [149].

Other studies reported the action of flavones in inhibiting autophagy. Baicalein (30 µM) was able to inhibit autophagosome formation stimulated by mTOR inhibition in stem cell-like cells (TICs) isolated from mouse and human liver tumors. Particularly, baicalein inhibited guanosine triphosphate (GTP) binding of SAR1B GTPase which is important for autophagic process, thus leading to cell death [132]. Moreover, nobiletin (40 μM), a polymethoxyflavonoid found in citrus fruits, suppressed the growth of SKOV-3/TAX paclitaxel-resistant human ovarian adenocarcinoma cells, by activating apoptosis, by inducing cell cycle arrest and by inhibiting autophagy. Nobiletin impaired the autophagic flux in these cells and in this way enhanced nobiletin-inducing apoptosis [150]. Similarly, another study reported the same effect of nobiletin (12.5–50 μM) in human gastric cancer SNU-16 cells [151]. Likewise, Toton et al. showed how increasing the concentration of zapotin, a natural flavonoid from the tropical fruit zapote blanco (30 μM), inhibited the formation of autophagosomes and decreased LC3 protein levels in HeLaPKCeA/E cancer cells which constitutively overexpressed the active protein kinase C epsilon (PKC_ε_) [152]. The combined treatment with luteolin (20 µM) and silibinin (50 µM) suppressed the autophagic activity, as demonstrated by the downregulated expression of LC3 I, LC3 II and Beclin 1, and induced the apoptotic process in U87MG and T98G glioblastoma cells [138]. Similarly, vitexin, apigenin-8-C-D-glucopyranoside (100 µM), inhibited autophagy to induce apoptosis through the JNK MAPK pathway in SK-Hep1 and Hepa1-6 HCC cells [153].

#### 3.1.4. Anthocyanins

Delphinidin is an anthocyanidin monomer with strong antioxidative capability present in vegetables and fruits. Different studies demonstrated its role in inducing cytoprotective autophagy in cancer cells. Indeed, delphinidin induced the formation of autophagic vacuoles, the conversion of LC3 I into LC3 II and increased the expression of the ATG5-ATG12 conjugate complex in HER-2^+^ breast cancer cell lines (MDA-MB-453, 80 µM; BT474, 140 µM). The use of autophagy inhibitors (3-MA, bafilomycin A1) increased the induction of apoptosis and the inhibition of cell proliferation, thus suggesting the activation of a cytoprotective autophagy by delphinidin. The mechanism of its activity involved the inhibition of the Akt/mTOR pathway and the activation of LKB1 and AMP [154,331]. Similarly, delphinidin (10–200 µM) induced autophagosomes, p62 degradation and the conversion of LC3 II in the human osteosarcoma cell line U2OS. Apoptosis was also observed after ROS induction and G0/G1 cell cycle arrest after applying the autophagy inhibitor 3-MA in these cells [156]. Delphinidin induced dose- (80–150 µM) and time-dependent autophagic vacuolization and induced the lipidated form of LC3 II in human HCC cell lines (SMMC7721, HCCLM3 and MHCC97L). In these cells, the inhibition of delphinidin-induced autophagy resulted in necrotic cell death, likely because of an ATP deficiency that prevented caspase activation and subsequent apoptosis [155]. Anthocyanidins (pelargonidin, cyanidin, malvidin, peonidin and delphinidin) (100 µM) induced autophagy in HeLa cervical cancer cells. Delphinidin (100 µM), in particular, increased the formation of the autolysosomes and autophagosomes. In addition, delphinidin induced protective autophagy in an ATG5-dependent manner, reducing the cytotoxicity in ATG5-deficient mouse embryonic fibroblasts [157].

Cyanidin-3-*O*-glucoside (C3G), the major anthocyanin identified in Chinese bayberry extract, protected pancreatic β cells from hydrogen peroxide (H_2_O_2_)-induced apoptosis and oxidative stress-mediated autophagic cell death. It activated autophagic flux and cell death in the rat pancreatic β cell line (INS-1) under oxidative stress conditions (H_2_O_2_ treatment). The pre-incubation of INS-1 cells with C3G (0.5–1 µM) decreased LC3 II generation and accumulation of autophagic vacuoles. In addition, autophagy also occurred in β cell grafts during the early phase post-transplantation in mice and anthocyanin preincubation decreased cell death in the graft [160]. C3G (80 µM) enhanced cytoprotective autophagy, by increasing the expression of autophagy-associated proteins ATG5 and LC3 II in UVA-exposed primary human dermal fibroblasts (HDFs). In this way, C3G decreased irradiation-induced oxidative stress and apoptosis [332].

Other studies reported the induction of cytoprotective autophagy by anthocyanins. Choe et al. reported that anthocyanins, extracted from black soybean (cv. Cheongja 3, Glycine max L.), induced autophagy, prior to the activation of apoptosis (100–300 µg/mL) in U2OS cells, by the activation of AMPK and MAPKs. In addition, anthocyanin-induced autophagy was prevented by inhibitors of AMPK, and not by inhibitors of ERK or Akt. The inhibition of AMPK also enhanced anthocyanin-induced apoptosis, thus suggesting a protective role for autophagy [164]. Cinnamtannin D1 (125, 150, and 175 μM) is an A-type procyanidin, isolated by *Rhododendron formosanum* extracts, that induced cell cycle arrest in the G1 phase and Beclin 1-independent autophagy, but not apoptosis, in NSCLC cells (A549, H460). Moreover, it activated autophagy by inhibiting the Akt/mTOR pathway and by activating the ERK1/2 pathway. However, the inhibition of autophagy amplified cell death, thus suggesting a cytoprotective role for cinnamtannin D1-induced autophagy [167].

The treatment of human colon cancer HT-29 cells (100 µg/mL) with Illawarra plum extract, containing anthocyanin-rich phenolics, resulted in the alteration of cellular morphology, although cells maintained their viability. Most cells presented cytoplasmic vacuoles and a trend for increasing sirtuin 1 (SIRT1) expression, which is necessary for starvation-induced autophagy. The extract also increased biomarkers of genotoxic damage and chromosomal instability. Nuclear buds were found inside the vesicles, a condition termed “piecemeal microautophagy of the nucleus” and they were associated with the accumulation of cells in the S phase of the cell cycle [166].

Another study reported a role for cyanidin in increasing the chemosensitivity of renal cell carcinoma (RCC) cells to cisplatin treatment, consecutive to autophagy impairment. Cyanidin (25–100 µM) inhibited 786-O and ACHN RCC cell proliferation, by cell cycle arrest and induction of apoptosis. Cyanidin also decreased oxidative stress-induced autophagic cell death in RCC cells [159].

On the other hand, several studies demonstrated the induction of autophagy as a cell death mechanism by different anthocyanins. Pelargonidin, an anthocyanin that is biosynthesized from flavonoid precursors and is responsible for the color of several fruits and flowers [333], exerted an antiproliferative effect mediated by autophagy on osteosarcoma U2OS cells. When the autophagy inhibitor 3-MA was combined with pelargonidin (15–30 µM) the cell viability was restored. In addition, pelargonidin downregulated the PI3K/Akt pathway and induced a dose-dependent increase in ROS and a significant decrease in mitochondrial membrane potential (MMP) that support the autophagic process [158]. Cheng et al. showed the reduction in diethylnitrosamine (DEN)-induced liver carcinogenesis after treatment of rats with mulberry water extract (MWE). This extract, rich in polyphenol (MPE) content, comprising phenolic acid (5.12%), flavonoids (8.23%) and anthocyanins (5.61%), reduced liver tumor foci, serum aspartate aminotransferase (AST) and alanine aminotransferase (ALT), and the activity of the enzyme gamma-glutamyltransferase (γ-GT). MPE was able to in vitro induce apoptosis of p53-positive HepG2 cells and autophagy of p53-negative Hep3B cells, by the activation of AMPK pathway and by the inhibition of PI3K/Akt/mTOR signaling [161]. Moreover, by the modulation of the Akt/mTOR pathway, mulberry anthocyanins (10 µg/mL) induced apoptosis and autophagy-dependent cell death in thyroid cancer cells (SW1736 and HTh-7) [162]. Similarly, mulberry anthocyanins induced autophagy, increasing the LC3 II/LC3 I ratio and the expression of Beclin 1 in human gastric cancer cells (SGC-7901) [163]. The juice of the Italian Pelingo apple, rich in polyphenols (1.996 mg/mL) and anthocyanins (28.39 mg/mL), induced a G2-phase cytostatic effect in human breast cancer cells (MCF-7, MDA-MB-231) (2.5% v/v of Pelingo juice). In addition, it upregulated p21, inhibited ERK1/2 activity, increased LC3 II/LC3 I ratio and induced the cellular vacuolization typical of autophagic conditions [165]. Weh et al. investigated the level of expression of Beclin 1 in 115 esophageal adenocarcinoma patients’ biopsies, reporting the loss of its expression in half of the specimens and its progressive reduction with advanced grades and stages of disease. Therefore, the authors employed proanthocyanidin-rich cranberry extract (C-PAC, 75 μg/mL) on esophageal adenocarcinoma cell lines (JHAD1, OE19) and found a modulation of autophagy by C-PAC. In fact, it reduced Beclin 1 level and induced Beclin 1-independent autophagy, which was associated with cell death [168].

#### 3.1.5. Flavanones

Flavanones have the potential to modulate autophagy, as shown by different studies. The chemopreventive effects of hesperidin against colon carcinogenesis were demonstrated in an azoxymethane (AOM)-induced mouse model. Hesperidin administration (25 mg/kg, oral), prior to or after AOM injection, inhibited PI3K/Akt/GSK-3β and mTOR pathways and activated apoptosis and autophagy, as shown by the increase in pro-apoptotic proteins, Beclin 1 and LC3 II in colonic tissues [169]. Conversely, naringin (50 and 100 mg/kg/d, oral, one week after AOM, for 8 weeks) prevented AOM/dextran sulfate (DSS)-induced colorectal inflammation and carcinogenesis, by inhibiting ER stress-mediated autophagy in mice [170]. The suppression of autophagy, via activation of PI3K/Akt/mTOR pathway, and the induction of apoptosis during endoplasmic reticulum stress was also demonstrated for pinocembrin in vitro (B16F10 and A375 cells; 50–150 µM) and in vivo (50 mg/kg or 75 mg/kg, i.v., daily for 14 days) in melanoma [172]. However, naringin (2 mM) was also able to induce autophagy, as shown by the formation of cytoplasmic vacuoles and autophagosomes, through the activation of Beclin 1 and LC3 II in human AGS gastric cancer cells. The activation of autophagy by naringin inhibited cancer cell growth and it was achieved through the downregulation of the PI3K/Akt/mTOR pathway via activation of MAPKs [171]. Thus, the effects of naringin on autophagy appear to be dependent on the type of cancer.

Other flavanones induced a protective autophagy. 5-Methoxyflavanone (5-MF; 40 µM), with high bioavailability and metabolic stability, promoted ERK-mediated autophagy in human colon cancer HCT-116 cells, which acted as a survival program against caspase-2 mediated apoptosis [173]. The induction of cytoprotective autophagy was also demonstrated for 6-*C*-(*E*-phenylethenyl)naringenin (6-CEPN) (10 µM for 24 h) in human colon cancer cells (SW620 and HCT-116) by Zhao et al. They showed that the blockade of autophagy led to enhanced necrotic cell death [174]. Conversely, 2′,3′-dimethoxyflavanone (2′,3′-DMF; 50–100 µM), which inhibited the growth of MCF-7-SC breast cancer stem cells through the activation of apoptosis, also induced the conversion of LC3 I into LC3 II, but it did not induce autophagic flux. Interestingly, the results showed that LC3 conversion mediated the accumulation and activation of the apoptosis initiator caspase-8 and thus enhanced apoptosis [334].

Flavanones were also employed in combination with anticancer agents. Liquiritin, one of the main flavonoids in licorice, was employed in combination with cisplatin (DDP) in DDP-resistant human gastric cancer SGC-7901/DDP cells. Liquiritin enhanced the sensitivity of cells to cisplatin exposure, by inducing apoptosis and autophagy in vitro (80 µM) and in vivo (15 mg/kg liquiritin, 3 mg/kg DDP or the two in combination i.p. daily), as shown by the increase in Beclin 1, LC3 II expression and by the reduction in p62 in cells and in gastric tumor tissues [175]. The flavanone silibinin (100 µM) suppressed the growth of prostate cancer cells (DU145) in the presence of the therapeutic agent arsenic, by reducing the arsenic-caused oxidative cell stress and by increasing arsenic-inducing cell death via autophagy and apoptosis. Thus, silibinin could be useful to sensitize prostate cancer cells to cell death during arsenic treatment [176].

#### 3.1.6. Isoflavones

Several studies investigated the modulation of autophagy by genistein. Gossner et al. reported that genistein (25–100 µM) induced apoptosis and a caspase-independent cell death with features of autophagy in ovarian cancer (A2780, CaOV3, and ES2) cells [177]. The genistein-antiproliferative effects was also demonstrated in MCF-7 breast cancer cells (100 µM), through the activation of apoptotic and autophagic cell death [178]. In addition, Pons et al. reported that genistein affected the efficacy of the anticancer therapies depending on the ERα/ERβ ratio in breast cancer cells (MCF-7, T47-D, MCF-7 overexpressing ERβ). They showed that the treatment with genistein (1 µM) combined with cisplatin or tamoxifen in cells with high ERα/ERβ ratios resulted in an increased cell viability due to the reduction in apoptosis and autophagy [335]. Conversely, it was reported that prepubertal and lifetime genistein consumption improved the sensitivity of mammary tumors to tamoxifen therapy, by reducing autophagy-related genes (GRP78, IRE1α, ATF4 and Beclin 1) in (9,10-dimethylbenz[a]anthracene (DMBA)-induced mammary tumors in female Sprague–Dawley rats fed with AIN93G diet supplemented with 500 ppm genistein) [179].

Genistein (60 µM) enhanced the radiosensitivity of NSCLC A549 cells by inducing apoptosis and autophagy. The mechanism of action involved a reduction in the cytoplasmic Bcl-xL levels, the increase in LC3 II, a decrease in p62 and the dissociation of Bcl-xL/Beclin 1 proteins. In addition, it was found that the stimulation of autophagy was necessary for the induction of apoptosis [180]. Similarly, Suzuki et al. reported that genistein activated apoptosis and autophagy to enhance the anticancer effects of 5-fluorouracil (5-FU) in human pancreatic cancer cells (MIA PaCa-2; 100 µM) and in a murine xenograft model (genistein 1.3 mg i.p. and 5-FU, every 4 days for 21 days) [181].

Other studies showed the inhibition of autophagy by genistein. Nazim et al. demonstrated autophagy by genistein in TRAIL-resistant human adenocarcinoma A549 cells. They showed that genistein (10–40 µM, alone or prior to TRAIL protein addition) induced the accumulation of LC3 II and p62 proteins, resulting in the inactivation of autophagic flux and thus enhancing TRAIL-induced tumor cell death [182]. Moreover, it was reported that the combination of indol-3-carbinol (I3C; 300 µM), from cruciferous vegetables, and genistein (40 µM) inhibited the survival of human colon cancer HT-29 cells by inducing apoptosis and autophagy through the downregulation of Akt and mTOR. However, the maturation of autophagosomes was inhibited by the combined treatment [183].

The effects of other isoflavones on autophagy were evaluated. Puerarin (100 µM) promoted apoptosis and autophagy and thus inhibited cell survival of K562 chronic myeloid leukemia (CML) cells. In this context, the use of the inhibitor 3-MA showed that the induction of apoptosis by puerarin was dependent on the activation of the autophagic flux in K562 cells [184]. Puerarin (20 µM) also inhibited the growth of NSCLC (NCI-H441) cells by inducing apoptosis and autophagy. The mechanism of action involved the inactivation of PI3K/Akt and ERK pathways [185,336].

NV-128 (0.1–10 µg/mL), an isoflavone derivative, was demonstrated to be able to induce autophagy in paclitaxel- and carboplatin-resistant epithelial ovarian cancer cells (EOC; R182), as demonstrated by the increase in LC3 II protein 8 h after treatment. However, the results also showed that the activation of autophagy was not the primary mechanism involved in the cell death observed after the NV-128 treatment [186].

Furowanin A is an isoflavonoid compound extracted from the leaves of *Millettia pachycarpa* Benth. Furowanin A (2 and 5 µM) promoted autophagy in HT-29 and SW480 colorectal cancer cells, as shown by the formation of AVOs, the increase in Beclin 1 and LC3 II, the decrease in p62 and the increase in autophagosome numbers. The use of 3-MA showed that the induction of autophagy by furowanin A promoted cell cycle arrest and protected colorectal cancer cells from apoptosis [187]. Another isoflavone, glabridin (1–100 µM), showed cytotoxic effects on human hepatoma cells (Huh7 cells) through the induction of apoptosis and autophagy. Glabridin treatment induced the formation of AVOs in cells and the increase in LC3 II and Beclin 1 protein expression. The use of autophagy inhibitors enhanced cell apoptosis, suggesting that glabridin-induced autophagy had a protective effect on liver cancer cells and occurred earlier than apoptosis [188]. Similarly, several studies have demonstrated that celastrol, from the Chinese herb *Tripterygium wilfordii*, induced autophagy, which promoted cell survival in different types of cancer cells. Celastrol (1.2 µM) induced autophagy, as a mechanism of cell survival, in HeLa cells, in A549 cells and in PC-3 cells derived from the cervix, lungs and prostate, as detected by the formation of autophagosomes and the change in LC3 protein [189]. Deng et al. also showed the activation of autophagy by celastrol (500 nM, prior to exposure to rotenone) to protect human neuroblastoma SH-SY5Y cells from rotenone-induced cell injury [190].

Conversely, Miyamoto et al. reported that phenoxodiol, a synthetic analogue of the plant isoflavone genistein with an improved anticancer efficacy (0.5–2 µg/mL), inhibited autophagy and X-linked inhibitor of apoptosis protein (XIAP), thus sensitizing ovarian clear cell carcinoma cells (KK cells) to cisplatin treatment [191].

### 3.2. Non-Flavonoids

#### 3.2.1. Coumarins

Coumarins have been shown to reduce the viability of cancer cells by modulating the autophagy in several studies. The activation of apoptosis, as well as a nonprotective autophagy and the generation of autophagic flux, mediated by the inhibition of the PI3K/Akt/mTOR pathway, were demonstrated for a novel hybrid of a 3-benzyl coumarin seco-B-ring derivative and phenylsulfonylfuroxan (50 nM) in NSCLC A549 cells [192]. Conversely, another hybrid compound of coumarin and phenylsulfonylfuroxan (200 nM) activated apoptosis and cytoprotective autophagy via the Akt/mTOR pathway, which rescued NSCLC cells (A549 and H1299) from death [193]. In the same cancer cells resistant to etoposide (A549RT-eto), feroniellin A (FERO; 0.05–1 mM), a novel furanocoumarin, induced autophagy, characterized by the conversion of LC3 I, the induction of GFP-LC3 puncta structures, the increase in Beclin 1 and ATG5 expression and the inhibition of mTOR. It was also observed that the induction of autophagy enhanced FERO-induced apoptosis; thus, it was not protective [194]. Esculetin (20 µM) was also demonstrated to be able to suppress the proliferation of human leukemia HL-60 cells, through the induction of apoptosis, autophagy, and the arrest of the cell cycle and the Raf/Mitogen-activated protein kinase/ERK kinase (MEK)/ERK signaling pathway [195]. Similarly, xanthoxyletin (5–20 µM) inhibited the growth of SCC-1 cells by modulating MEK/ERK pathway, and by inducing apoptosis, autophagy and cell cycle arrest [196]. Recently, it was shown that osthole (7-metoxy-8-isopenthenocoumarin; 150–250 µM), alone and with TMZ, triggered autophagy in human glioblastoma multiforme (T98G) and anaplastic astrocytoma (MOGGCCM) cells, although the main type of induced death in these cells was apoptosis [197]. Two major coumarins extracted from *Psoralea corylifolia* (50–400 µg/mL), psoralen and isopsoralen, exerted a cytotoxic effect in prostate cancer cells PC-3, through the induction of apoptosis and autophagy [198]. The induction of autophagic cell death in prostate cancer cells (PC-3 and DU145) was also demonstrated for a geranylated 4-phenylcoumarin (DMDP-1; 9 µM), extracted from the bark of *Mesua elegans* (Clusiaceae) [199]. Recently, Cui et al. reported that hydroxypyridinone-coumarin (2 µM) induced autophagy and thus inhibited the proliferation of HCC cells (MHCC97 and HepG2) by activating ERK1/2, by inhibiting Akt, by increasing ATG5, ATG3, Beclin 1 and LC3 II proteins and by reducing p62 levels [200]. Similarly, Li et al. reported the induction of apoptosis and autophagic cell death by psoralidin (9–26 µM) leading to the inhibition of the liver cancer cell line HepG2 proliferation, as shown by the presence of autophagosomes, increase in LC3 II and Beclin 1 expression [201]. In addition, the coumarin-derivate compound 5-methoxypsoralen (Bergapten) upregulated PTEN and p38 MAPK/NF-Y pathways and inactivated Akt/mTOR pathway resulting in autophagy and the inhibition of the survival of breast cancer cells (MCF-7 and ZR-75). Bergapten increased the expression of Beclin 1, PI3KII, UV radiation resistance associated gene protein (UVRAG), autophagy and Beclin 1 regulator (AMBRA), the conversion of LC3 I into LC3 II and the formation of autophagosomes [203]. Conversely, Ren et al. showed that psoralidin (2.5–10 µM) induced DNA damage and protective autophagy mediated by NADPH oxidase 4 (NOX4) in MCF-7 breast cancer cells [202].

#### 3.2.2. Curcuminoids

The effects of curcumin on the modulation of autophagy were investigated in different cancers. Curcumin (5–40 µM) showed antiproliferative activity by inducing apoptosis and autophagy in human NSCLC cell line A549. Autophagic vesicles, presence of double membrane-enclosed structures, an increase in Beclin 1, LC3 II expression, and LC3 II/LC3 I ratio and a decrease in p62 were detected upon curcumin treatment. Moreover, curcumin induced apoptosis and autophagy by the inhibition of PI3K/Akt/mTOR signaling [204,205]. Further investigations confirmed that curcumin (40 µM) triggered autophagy in A549 cells through the activation of the AMPK signaling pathway, an increase in gene expression levels of human ganglioside (GD)-3 synthase (hST8Sia I) and of its catalyzed protein product GD [206]. Similarly, Wang et al. reported that curcumin (10 µM) inhibited the growth of other NSCLC cells (H1299; A549), it suppressed PI3K/Akt/mTOR activation and thus it induced apoptosis and autophagy [207]. Moreover, curcumin (15 µM) enhanced the autophagy induced by galbanic acid (40 µM), a sesquiterpene coumarin in NSCLC A549 cells. A549 cells were also pre-treated with rapamycin (mTOR inhibitor) or insulin (Akt activator) to demonstrate the involvement of the Akt/mTOR pathway in mediating the anticancer effect [208].

Curcumin (10–80 µg/mL) was also demonstrated to be able to induce cell cycle arrest in the G2/M phase, apoptosis and autophagy, by reducing mTOR protein expression in human pancreatic cancer cell lines (PANC1, BxPC3). The results showed an increase in LC3 II, autophagosomes and LC3-puncta formation upon curcumin treatment [210].

Gastric cancer cells (SGC-7901, BGC-823) exposed to curcumin (10–40 µM) showed the inhibition of cell proliferation and a dose-dependent increase in apoptosis. Autophagy was also activated by curcumin, as shown by the increase in Beclin 1, ATG5 and ATG3 protein levels, and the decrease in LC3 I converted to LC3 II [211]. Li et al. evaluated the effects of curcumin in the same gastric cancer cell lines and in the MKN-28 cell line. Curcumin (5–20 µM) suppressed cell growth and induced apoptotic cell death and autophagy in vitro. The inhibition of autophagy enhanced the curcumin-induced cell death, thus suggesting a cytoprotective role for autophagy and new strategies for the treatment of this tumor [212]. The induction of a protective autophagy was also reported in human ovarian cancer cell lines (SKOV-3, A2780, HO-8910). Curcumin (10–40 µM) induced apoptosis and autophagy, as shown by the formation of autophagic vesicles, the increase in LC3 I/II, ATG3, Beclin 1 expression and LC3-puncta formation. When combined with chloroquine, late autophagic steps were suppressed and apoptosis increased in ovarian cancer cells [213].

Other studies demonstrated the activation of autophagy by curcumin in colon cancer cells [214,337]. In particular, the contribution of HSP27 in the apoptotic and autophagic processes induced by curcumin (20 µM) was demonstrated in HT-29 and DLD-1 colon cancer cells. Indeed, the two cell lines showed HSP27 expression, and the sensitivity to curcumin cytotoxicity was correlated with the high expression level of this protein [337]. The involvement of yes-associated protein (YAP) in the regulation of the autophagic flux induced by curcumin in colon cancer cells (SW620, HCT-116) was demonstrated by Zhu et al. Indeed, curcumin (10–30 µM) induced moderate cytotoxicity, an increase in LC3 protein, a decrease in p62 expression and led to a reduction in YAP expression [214]. On the other hand, pre-treatment with curcumin enhanced the cytotoxicity of the anticancer alkylating agent 5-FU in vitro and in vivo in colon cancer. In particular, this effect was achieved through the inhibition of 5-FU-induced autophagy by curcumin (10–30 µM) in colon cancer cells (HCT-116, HT-29). Autophagy signaling pathway was also modulated and phospho-Akt, phospho-mTOR, phospho-AMPK and phospho-ULK1 were downregulated. These results were confirmed in vivo in xenograft mice [215].

Furthermore, it has been shown that curcumin modulates autophagy in HCC cells. Curcumin (5–20 µM) reduced cell proliferation and increased the apoptosis in vitro (HepG2 cells) and in a xenograft mouse model. In addition, curcumin inhibited glypican-3 (GPC3)/Wnt/β-catenin pathway through the activation of autophagy [216]. Conversely, Elmansi et al. demonstrated the in vivo hepatoprotective effects of curcumin-induced autophagy in a rat model of thioacetamide (TAA)-induced HCC. Curcumin (100 or 200 mg/kg, oral, daily) increased LC3 II and Bcl-2 gene expression levels and decreased p62/Sequestosome-1 (SQSTM1) level. Thus, curcumin activates autophagy and at the same time switches off apoptosis [217].

Recently, a study investigated the role of the JNK-associated leucine zipper protein (JLP) in curcumin-induced cell death in cancer cell lines (cervical cancer HeLa, colon carcinoma HCT-116, HCC HepG2). The results showed that JLP protected against curcumin-induced cell death (40 µM), by activating autophagy and p38 MAPK [218].

Deng et al. showed that curcumin (5, 20, 80 µM) exerted the opposite effect, depending on the dose, in RCC cell lines (786-O, ACHN). Curcumin increased LC3 II protein in a concentration-dependent manner, but phospho-AMPK and proteins of the endoplasmic reticulum stress pathway (GRP78 and CHOP) were increased at low doses of curcumin and diminished at high doses of curcumin treatment. Finally, intracellular ROS were significantly elevated at high doses of curcumin but lower than the control when applying low doses of curcumin. These contrasting results reveal that autophagy is activated to protect cells from a stressful condition, but when the insult is highly potent, autophagy is activated to kill the cells and to avoid dangerous consequences [219].

Our group demonstrated that curcumin (25 µM) inhibited malignant mesothelioma cell proliferation, both in human (MM-B1, H-Meso-1, MM-F1) and in murine (#40a) cell lines, by increasing ROS production and thus inducing DNA damage. In addition, curcumin triggered autophagy, but the autophagic flux was blocked, as revealed by the cytoplasmic accumulation of p62 protein, and was coincident with caspase-8 activation which led to apoptosis [220]. We also demonstrated that induction of autophagy by curcumin is important to stimulate antitumor immune responses in breast cancer, and that autophagy inhibition by chloroquine reduced such responses. In fact, curcumin (25 µM) induced a complete autophagic flux that played a pro-survival role in murine Her2/neu positive breast cancer cells (TUBO). Indeed, chloroquine increased the anticancer effects of curcumin in vitro and in nude mice. However, when chloroquine was administered in combination with curcumin in immunocompetent mice-bearing TUBO cells, it completely inhibited the anticancer effects of curcumin, because of the recruitment of regulatory T cells in the tumor microenvironment in mice [221]. Moreover, we also reported that combined treatment with curcumin and resveratrol was more effective in inhibiting in vitro and in vivo HNSCC cell growth than the treatment with curcumin alone. Indeed, the combined treatment enhanced the apoptotic effect of curcumin and more actively stimulated the formation of double membranes surrounding vast portions of cytoplasm, which was mediated by the inhibition of Akt phosphorylation in HNSCC cells (FaDu, CAL-27) [222].

It was reported that curcumin promoted early cytotoxic effects, independently of apoptosis, in the Philadelphia chromosome-positive acute lymphoblastic leukemia (Ph^+^ ALL) cell line (SUP-B15). Curcumin increased the LC3 II/LC3 I ratio, thus triggering autophagic cell death, by activating the Raf/MEK/ERK pathway [223]. Similarly, cell death and autophagy were induced by curcumin (10 µM) when used to treat the glioblastoma cell line A172. LC3 II and LC3-puncta structures increased together with autophagy proteins ATG5, ATG12 and Beclin 1. Autophagy contributed to curcumin-mediated cell death [224]. Natural curcumin (25 µM) or solid lipid curcumin particles (SLCP; 25 µM) were also demonstrated to be able to induce autophagy in other glioblastoma cell lines (human, U87MG; mouse, GL261; rat, F98), rat glial tumor cells (C6-glioma) and mouse neuroblastoma cells (N2a cells). In particular, the treatments modulated autophagic markers (ATG5, ATG7, Beclin 1, LC3) and CMA markers (LAMP-2A, HSP70, HSP90), decreased mitophagy markers and inhibited the Akt/mTOR pathway. The formation of autophagic vacuoles, membrane blebbing, cytoskeleton disorientation and chromosomal condensation were greatly increased by SLCP in the U87MG cell line, compared to curcumin treatment [227].

Other investigations revealed the effects on autophagy of curcumin in combination with other compounds or therapies. Curcumin (20 µM), combined with the mTORC1/2 inhibitor PP242, induced cell death in human cancer cells (renal carcinoma: Caki, ACHN, A498; glioma: U87MG; breast carcinoma: MDA-MB-231), by downregulating Rictor and Akt protein levels. The inhibition of Rictor increased cytosolic calcium release, resulting in lysosomal damage and the induction of autophagy. Pre-treatment with the autophagy inhibitor 3-MA inhibited the apoptosis induced by PP242 and curcumin, suggesting that this combined treatment activated autophagy and secondarily apoptosis [225].

Curcumin (2 μM) combined with sildenafil (2 μM), the phosphodiesterase 5 inhibitor, showed a synergic cytotoxic interaction and the activation of the intrinsic and extrinsic pathways of apoptosis in gastrointestinal cancer cell lines (HCT-116, HT-29, HepG2, Huh7). The autophagic flux was also activated. The knockdown of molecules involved in autophagy, ATG5 or Beclin 1, reduced the frequency of dead cells after treatment, and similar effects were obtained using the autophagy inhibitor chloroquine or 3-MA. Conversely, the impairment of autophagosomes formation, through the knockdown of ATG16-L1, increased the cell death induced by curcumin plus sildenafil treatment [226].

In another report, curcumin was combined with photodynamic therapy (PDT), a new method to kill damaged cells or unwanted tissues. In particular, the cytotoxic effects of curcumin-layered double hydroxide (LDH) nanohybrid (25 and 100 µg/mL) after PDT (blue light LED irradiation) were evaluated in the human breast cancer cell line MDA-MB-231. The treatment inhibited cell proliferation and induced autophagy, apoptosis and cell cycle arrest [228].

Several studies evaluated the effects of curcumin metabolites, analogs or derivatives.

It has been reported that curcumin or the metabolite tetrahydrocurcumin induced cell death in chemotherapy-resistant (Ara-C) human AML cells (HL-60) with two different modalities. In fact, curcumin mainly activated apoptotic cell death, through the regulation of poly(ADP-ribose) polymerase (PARP), caspase-9 and caspase-3, while tetrahydrocurcumin mainly induced autophagy by increasing both LC3 and p62 [231]. Tetrahydrocurcumin (10–130 µM) was also employed in human NSCLC A549 cells. The treatment inhibited cell growth and activated the autophagic flux by inhibiting the PI3K/Akt/mTOR pathway and increasing the gene expression of Beclin 1 [232].

The curcumin analog (3E,5E)-3-(3,4dimethoxybenzylidene)-5-[(1H-indol-3-yl)methylene]-1-methylpiperidin-4-one (CA-5f) induced dose- and time-dependent cytoplasmic vacuolization, LC3 II level increases and LC3-puncta formation both in NSCLC cells (A549) and human umbilical vein endothelial (HUVEC) cells. In addition, CA-5f increased the protein level and the recruitment of p62/SQSTM1 to phagophores, suggesting that CA-5f inhibited autophagic flux instead of enhancing it. Similar effects were demonstrated in two other NSCLC cell lines (H1299, H157), in a human HCC cell line (HepG2), in a human cervical cancer cell line (HeLa) and in human embryonic kidney 293 cells (HEK293). Further experiments confirmed that CA-5f played a role as a late-stage autophagy inhibitor. When administered in vivo in nude mice bearing A549 cells, CA-5f (40 mg/kg, i.v., every two days) suppressed tumor growth, inhibited autophagic flux and induced apoptosis [233]. A549 cell line was also employed to evaluate the activation of the autophagic flux with the chemically synthesized curcumin derivative ZYX01. The compound induced autophagy through AMPK/ULK1/Beclin 1 pathway and increased the LC3 II/LC3 I ratio, upregulated Beclin 1 and downregulated p62 expression [234]. Similarly, the activity of the novel curcumin derivative, 2-(3-{(1E)-{(E)-3-(4-hydroxy3-methoxybenzylidene)-2-oxocyclohexylidene)methyl)-1H-indol-1-yl)acetic acid} (MOMI-1), was evaluated in A549 cells. MOMI-1 (20 µM) inhibited cell proliferation by inducing autophagy [235].

The cytotoxic effects of Bis(hydroxymethyl) alkanoate curcuminoid derivative (MTH-3), a curcumin derivative, were evaluated (10 µM) in a human breast adenocarcinoma cell line (MDA-MB-231). MTH-3 induced the inhibition of proliferation, G2/M phase cell cycle arrest, a reduction in CDK1 kinase activity, and the induction of apoptosis. In addition, MTH-3 stimulated autophagy by increasing LC3B and p62 expression in double immunostaining experiments, and by increasing LC3B, ATG5, ATG7, ATG12, and Beclin 1 protein levels [236]. A human oral cancer cell line (SAS) was treated with curcumin, and its derivatives demethoxycurcumin (DMC) and bisdemethoxycurcumin (BDMC). All compounds, to different extents, induced autophagy, apoptosis and a reduction in cell viability. Curcumin (30 μM) and DMC or BDMC (15 μM) increased the number of autophagic vacuoles, and modulated autophagy-associated proteins. Pretreatment with 3-MA, in combination with curcumin and its derivatives, increased cell viability, suggesting that autophagy enhanced curcuminoid-induced apoptosis in oral cancer cells [229]. Autophagy and apoptotic cell death induction were evaluated on the SAS cell line, combining the three curcumin-based compounds (curcumin, DMC, BDMC) with gefitinib, the epidermal growth factor receptor (EGFR) tyrosine kinase inhibitor. The results showed that apoptotic cell death was greatly enhanced by the combined treatment (gefitinib 40 μM, plus curcumin 20 μM, DMC 5 μM or BDMC 5 μM). The co-treatment increased the autophagic vacuoles and the expression of autophagy-associated proteins (ATG5, LC3, Beclin 1, p62/SQSTM1, ULK1, Vps34). In addition, the in vivo administration of gefitinib with curcumin, DMC or BDMC (30 mg/kg each, i.p., every two days) in SAS xenograft nude mice demonstrated that gefitinib co-administered with curcumin and DMC, but not with BDMC, induced a significant reduction in tumor growth in mice, compared to single treatments. Tumor tissue analysis showed the increased expression of caspase-6 and -7, associated with apoptosis, and of Beclin 1 and LC3, associated with autophagy, by the combination of gefitinib with curcuminoids [230]. The autophagic activation induced by the combined treatment of curcumin and gefitinib was also evaluated in gefitinib-resistant NSCLC cells H157 and H1299. Curcumin (10 μM) enhanced the inhibitory effect of gefitinib, by inhibiting EGFR activity. Compared to the single treatment, the combination resulted in a greater increase in autophagic flux (increase in LC3 II puncta and AVOs), the accumulation of LC3 II and a reduction in SQSTM1. The co-treatment also induced autophagy-mediated apoptosis. Curcumin combined with gefitinib greatly reduced tumor growth, through the induction of autophagy-related cell death in nude mice bearing H157 or H1299 tumor cells [209].

The effects of a novel curcumin derivative, WZ35, were evaluated in HCC cells (HCCLM3), compared to curcumin treatment. WZ35 induced cell death and apoptosis and downregulated YAP signaling. In addition, curcumin induced autophagy, while WZ35 exerted an opposite effect on autophagy, by reducing LC3 I/II, ATG7 and Beclin 1 and by accumulating p62. Additional experiments demonstrated that YAP downregulation contributed to the autophagy inhibition caused by WZ35. When administered to BALB/c nude mice bearing HCCLM3 cells, WZ35 (20 mg/kg, i.p.) suppressed tumor growth more efficiently than curcumin and large areas of necrosis were present in treated tumors [237].

#### 3.2.3. Phenolic Acids

Hydroxybenzoic acid derivatives have been studied for their role in modulating autophagy and cancer cell growth. Duan et al. reported that ellagic acid (10–50 µM) activated autophagy, but not apoptosis, in lung cancer cells (HOP62 and H1975), and in tumor-bearing mice (40 mg/kg, i.p., every 2 days, for 22 days), as demonstrated by the formation of LC3-positive autophagosomes, the increase in LC3 II, ATG5 and the reduced levels of p62 [238]. Similarly, ellagic acid (36.6 µM) inhibited cell growth and the invasiveness of ovarian cancer SKOV-3 cells and stimulated apoptosis by activating cytotoxic autophagy, as indicated by the increase in the levels of Beclin 1, ATG5, LC3I/II and decrease in p62 [239]. In addition, it was shown that ellagic acid activated autophagy through the downregulation of mTORC1 and Akt, and the activation of AMPK [238,239]. The activation of apoptotic and autophagic cell death was also reported for punicalagin, an ellagitannin isolated from the fruit of *Punica granatum* L. trees. Punicalagin (1–30 µg/mL) increased LC3 II cleavage and the formation of autophagosomes in human U87MG glioma cells, by the activation of AMPK and p27 [240]. Recently, it was also shown that *Grias nuberthii* extract (20–50 µg/mL), which contains lupeol, 3′-*O*-methyl ellagic acid 4-*O*-*β*-D-rhamnopyranoside, and 19-*α*-hydroxy-asiatic acid monoglucoside, inhibited the growth of human colon cancer (RKO and SW613-B3) cells by inducing autophagy only (increase in Beclin 1, LC3 II and decrease in p62) [241]. It was also reported that ellagic acid (10–100 µM) inhibited the growth of human ovarian cancer cells (ES-2 and PA-1) by blocking the cell cycle at the G1 phase and by inducing apoptosis. However, it was shown that the simultaneous treatment of these cells with doxorubicin, paraplatin or paclitaxel, resulted in the suppression of drug-induced autophagy by ellagic acid in ES-2 cells. Thus, ellagic acid could be a useful tool to enhance the sensitivity of ovarian cancer cells to chemotherapeutic agents [338].

Gallic acid, the main component of *Terminalia bellirica* extract (0.1, 0.5, 1 mg/mL), showed anti-proliferative activity in OSCC Cal33 cells and induced mitochondrial apoptosis and autophagy, but this latter process was incomplete, as demonstrated by the increase in p62 protein and attenuation of autolysosome formation. Moreover, it was shown that the inhibition of autophagy increased the induction of apoptosis by this extract [242]. The induction of cytoprotective autophagy, by the inhibition of the Akt/mTOR pathway, was also demonstrated for paeonol (0.6–1.2 mM), a phenolic acid compound isolated from the Moutan Cortex in ovarian cancer cells. It was shown that the combination of paeonol and an autophagy inhibitor resulted in the enhancement of the suppression of cell viability and the induction of apoptosis in A2780 and SKOV-3 cells and in the A2780 xenograft model (40 mg/kg, i.p., every 2 days, for 12 days) [243]. Autophagy activation provided a survival signal which suppressed caspase-mediated and apoptotic deaths in prostate cancer cells (DU145, PC-3, TRAMP-C2) upon treatment with penta-*O*-galloyl-*β*-D-glucose (PGG). PGG (25–75 µM) induced the formation of autophagosomes and the lipid modification of LC3, with the inhibition of mTOR-downstream targets S6K and 4EBP1, and increased Akt activation [244]. Similarly, Xu et al. reported that corilagin [*β*-1-*O*-galloyl-3,6-(R)-hexahydroxydiphenoyl-D-glucose] induced the inhibition of gastric cancer cell (SGC-7901 and BGC-823) growth by activating apoptosis. Moreover, corilagin (10–30 µM) triggered autophagy, which had a cytoprotective effect [245].

Hydroxycinnamic acid modulated autophagy in cancer cells as well. The major constituent of propolis, CAPE (25 µg/mL), and the ethanol extract of Chinese propolis (EECP; 25–100 µg/mL) inhibited the LPS-stimulated MDA-MB-231 breast cancer cell growth by inducing apoptosis and autophagy [246]. Conversely, Yu et al. reported that CAPE (10 µM) induced a cytoprotective autophagy by AMPK activation in C6 glioma cells. The authors showed that CAPE inhibited the growth of C6 glioma cells and the use of a combination treatment with AMPK or autophagy inhibitors resulted in enhanced cytotoxicity [247]. Similarly, decyl caffeic acid caused protective autophagy in colorectal cancer cells. In particular, decyl caffeic acid (40 µM) suppressed the growth of HT-29 and HCT-116 cells, through the induction of cell cycle arrest and the blocking of the STAT3 and Akt pathway. Moreover, decyl caffeic acid induced a protective autophagy in HCT-116 cells by the increase in ATG3, ATG16, Beclin 1 and LC3 I/II proteins. The use of the autophagy antagonist 3-MA showed that the suppression of autophagy resulted in the enhancement of cell death by the induction of apoptosis [248]. Similarly, Endo et al. suggested that artepillin C, a cinnamic acid derivative in Brazilian green propolis, could be used in combination with autophagy inhibitors as a novel, complementary and alternative treatment for prostate cancer. They showed that artepillin C (50–100 µM) induced autophagy and apoptosis in CWR22Rv1 cells, and that co-treatment with autophagy inhibitors enhanced apoptosis and necroptosis [249]. Cinnamic acid, cinnamic aldehyde and coumarin, contained in *Cinnamomum cassia* extracts (50–100 µg/mL), induced autophagy as a survival pathway in human oral cancer cells (SASVO3). Indeed, co-treatment with the extracts and the autophagy inhibitor enhanced the suppression of cell viability and apoptosis [250].

The hydroxycinnamic acid ferulic acid (4-hydroxy-3-methoxycinnamic acid; 2.0–4.0 mM) had antiproliferative effects in human cervical cancer cells (HeLa and Caski) by inducing cell cycle arrest in the G0/G1 phase, apoptosis and by inhibiting autophagy [251]. Conversely, Pellerito et al. recently reported that a novel synthetic derivative of ferulic acid, tributyltin(IV) ferulate (400 nM) inhibited the proliferation of colon cancer cells (HCT-116, HT-29, Caco-2) by inducing G2/M cell cycle arrest and autophagic cell death, as shown by the formation of autophagic vacuoles and the increase in LC3 II and p62 [252]. The induction of apoptosis, as well as autophagy, as mechanisms for inducing cytotoxicity to neuroblastoma N2a cells, were also reported for *p*-coumaric acid (150–200 µM) [253]. Reis et al. studied the effects of the methanolic extract of *Ganoderma lucidum* (66.6 and 133.2 µg/mL), which contains *p*-hydroxybenzoic acid, *p*-coumaric acid and cinnamic acid, in the induction of autophagy in AGS gastric cancer cells. They showed an increase in autophagosome formation and LC3 II levels upon treatment with the extract, while a decrease in p62 cellular levels confirmed the autophagy induction in these cells [254].

#### 3.2.4. Lignans

Several studies demonstrated the ability of lignans to modulate autophagy. Honokiol, extracted from *Magnolia officinalis*, induced autophagy in different types of cancer cells, including melanoma, osteosarcoma, thyroid cancer, neuroblastoma and glioma [255,256,257,258,259,260]. Treatment with honokiol (30–40 µM) inhibited melanoma cancer cell (B16-F10, SKMEL-28) growth by inducing cell cycle arrest and autophagy with the formation of autophagosomes and an increase in LC3 II expression and cytoplasmic accumulation. The activation of autophagy was mediated by the inhibition of the Akt/mTOR pathway and Notch signaling [255,256]. A similar induction of autophagy as well as apoptosis was demonstrated for honokiol (10–20 µg/mL) in osteosarcoma cells (MG-63), through the inhibition of the PI3K/Akt/mTOR pathway [257] in human thyroid cancer cells (ARO, WRO, SW579; 20–60 µM) and in a xenograft nude mouse model (5 or 15 mg/kg, oral, every 3 days) [258]. Lin et al. also reported that honokiol (50 µM) induced autophagic apoptosis in neuroblastoma (neuro-2a and NB41A3) cells, through a p53-dependent pathway. Indeed, the use of the autophagy inhibitor 3-MA attenuated honokiol-induced autophagy and apoptotic cell death [259]. Similarly, it was shown that honokiol treatment (40 µM) enhanced the induction of autophagy and apoptosis induced by TMZ in drug-sensitive (human U87MG, murine GL261) and resistant (human U87-MR-R9) glioma cells, and that pre-treatment with autophagy inhibitors resulted in the attenuation of honokiol- and TMZ-induced cell autophagy and apoptosis [260].

Other lignans exerted cytotoxic effects by inducing autophagy in several cancer cells. For example, vitexin 6, from the seeds of *Vitex negundo* (5–20 µM), promoted autophagy and apoptosis, to induce cell death in breast (T-47D) and colon cancer (RKO) cells, through the activation of the JNK pathway [264]. A similar modality of action was reported for licarin A (10–25 µM), from the seeds of *Myristica fragrans*, which induced cell death in NSCLC cells (A549, NCI-H23), through the activation of both apoptosis and autophagy. The use of chloroquine showed that licarin A-induced autophagy played a death-promoting role in these cancer cells [265]. The induction of autophagy as a cell death mechanism was also reported for trachelogenin, magnolin and justicidin A in colorectal cancer cells [266,267,268]. Trachelogenin (5–10 µM), a lignan isolated from *Combretum fruticosum*, promoted autophagic cell death, but not apoptosis, as shown by the appearance of AVOs and autophagic vacuoles, by the increase in Beclin 1 expression and by the conversion of LC3 I into LC3 II in HCT-116 colon cancer cells [266]. Magnolin (10–40 µM) induced autophagy, which led to cell cycle arrest and the inhibition of cell growth (HCT-116 and SW480) in vitro, and in xenograft tumors (20 mg/kg, daily, i.p., for 33 days) [267]. Justicidin A (0.5–1.5 µM), isolated from *Justicia procumbens*, promoted autophagic flux, which enhanced apoptosis in human colorectal cancer cells (HT-29) and in xenograft tumors (6.2 mg/mouse/day, oral, for 56 days) [268]. Similarly, Ning et al. reported that pinoresinol inhibited, in vitro (10–40 µM) and in vivo (40 mg/kg, i.p., thrice a week, for 6 weeks), ovarian cancer cell (SKOV-3) growth by inducing autophagy, with the formation of autophagic vesicles, an increase in LC3 II and Beclin 1 expression, a decrease in p62 expression, and the inhibition of cell invasion and the RAS/MEK/ERK pathway [269].

The major lignan in sesame oil, sesamin, (50 µM) inhibited the proliferation of cervical cancer (HeLa) cells by inducing endoplasmic reticulum stress-mediated apoptosis and autophagy. The results showed an increase in autophagosomes, LC3 II and Beclin 1 and the decreased cytotoxic effects of sesamin after the inhibition of autophagy [270]. Moreover, sesamin (50 µM) also reduced the viability of colon cancer cells (HT-29, LS180) by inducing autophagy, but not apoptosis [271]. Similarly, magnolol (80 µM) induced cell death by autophagy, but not by apoptosis, in human NSCLC (H460) cells, through the downregulation of the Akt pathway [272]. Moreover, it was demonstrated that magnolol (10–20 µM) induced cell death by activating autophagy, via downregulation of the Akt/mTOR pathway, as well as apoptosis, in NSCLC cells (A549 and NCI-H1299) [273]. Conversely, Rasul et al. reported that magnolol (40–80 µM) induced autophagy in human gastric adenocarcinoma (SGC-7901) cells at high concentrations, but this mechanism was not involved in cell death, which was mainly mediated by the activation of apoptosis. Indeed, magnolol-induced cell death was not suppressed when the cells were co-treated with the autophagy inhibitor 3-MA [274].

Other studies investigated the role of arctigenin, isolated from the seeds of *Arctium lappa*, in modulating autophagy. It was reported that arctigenin (1–200 µM) induced autophagic cell death by inhibiting mTOR activation in ER^+^ breast cancer (MCF-7) cells [275]. Moreover, arctigenin (100 µM) enhanced the sensitivity of cisplatin-resistant colorectal cancer (R-SW480, R-SW620) by activating autophagy, which induced cell apoptosis and inhibited cell growth [276]. Recently, it was demonstrated that arctigenin inhibited autophagy. It was reported that arctigenin (1.25–10 µM) suppressed cell proliferation and blocked the autophagic pathway, as shown by the accumulation of p62 in HCC HepG2 cells [277].

Several studies reported the induction of autophagy as a cell survival mechanism by different lignans. The induction of apoptosis and a cytoprotective autophagy, mediated by ROS, was reported for honokiol (40 µM) in prostate cancer cells (PC-3, LNCaP, murine Myc-CaP) [261]. A similar effect was demonstrated with the combined treatment of honokiol and magnolol (20–40 µM, each) in glioblastoma cells (U87MG and LN229). The results showed the induction of autophagy and apoptosis, but autophagy played a protective role, as suggested by employing autophagy inhibitors, which enhanced the apoptosis mediated by the lignan treatment [263]. Recently, Kwon et al. also reported the same effect for a lignan ((−)-(2*R*, 3*R*)-1,4-*O*-diferuloylsecoisolariciresino (DFS); 10 µM) from *Alnus japonica*, which induced autophagy and endoplasmic reticulum stress in prostate and colon cancer cells (DU145, SW480). However, for this lignan, the autophagy inhibition also enhanced the cytotoxicity, suggesting that DFS induced autophagy for cell survival [278]. A double-edged sword role for autophagy was demonstrated for honokiol in OSCC by Huang et al. In this study, honokiol (20–40 µM) inhibited the growth of cancer cells (OC2, OCSL) by inducing apoptosis, cell cycle arrest and autophagy. In addition, co-treatment with the autophagy antagonist 3-MA fully decreased the viability of honokiol-treated cells. However, the enhancement of autophagy by rapamycin used in combination with honokiol also decreased cell viability. Thus, the authors suggested that a basal activity of autophagy could protect cancer cells from cellular stress, while its overactivation could cause cell death as well. The inhibition of cell growth by the induction of apoptosis and autophagy was also demonstrated in a nude mice xenograft model (5 and 15 mg/kg, oral, twice a week) [262].

#### 3.2.5. Stilbenes

Numerous studies have described the therapeutic and chemopreventive properties of resveratrol, also highlighting its role in the modulation of the autophagic process in many types of cancers [339,340].

Resveratrol induced autophagy-dependent apoptosis in the human promyelocytic leukemia cell line HL-60. Resveratrol treatment (12.5–100 μM) induces cell death by activating both the intrinsic and extrinsic pathways of apoptosis and by activating autophagy, as shown by the increase in the number of autophagosomes and LC3 II levels. Moreover, the activation of autophagy was mediated by LKB1-AMPK and PI3K/Akt/mTOR pathways [279]. Similarly, resveratrol (50 µM) triggered autophagic cell death in imatinib-sensitive and imatinib-resistant CML cells (K562) via JNK-mediated p62 expression and AMPK activation [280]. Miki et al. demonstrated that resveratrol (75–150 μM) induced apoptosis and the autophagic process in colorectal carcinoma cells (HT-29, COLO201) by increasing intracellular ROS levels. In addition, resveratrol treatment in the presence of 3-MA significantly reduced the percentage of apoptotic cells [281]. Likewise, resveratrol (12.5–100 μM) inhibited RCC cell (HK-2 and Ketr-3) growth by promoting apoptosis and autophagy, through the activation of AMPK, the inhibition of mTOR and the upregulation of autophagy-associated proteins [282]. In a similar way, resveratrol (30–50 μM) induced cell death through apoptosis and autophagy in several cervical cancer cells (C33A, CaLo, and HeLa) [284] and in the cisplatin-resistant human oral carcinoma cells (CAR) [285]. The activation of autophagy by resveratrol, as a cell death mechanism, was also demonstrated in HCC cells (MHCC-97H). Indeed, the use of 3-MA counteracted the inhibitory effect of resveratrol (20–100 μM) on HCC cell proliferation. Moreover, resveratrol induced the autophagic process by activating p53, while suppressing the PI3K/Akt pathway in HCC cells [283].

Different studies investigated the effect of resveratrol on autophagy in glioma cells. Yamamoto et al. demonstrated that resveratrol mediated anticancer effects in glioma cells by activating autophagy. Resveratrol inhibited the growth and induced the cell death of U373 human glioma cells and the presence of autophagosomes. Moreover, p38 and ERK1/2 promoted resveratrol-induced autophagy in glioma cells [286]. Conversely, a cytoprotective role for the autophagy induced by resveratrol in other glioma cells was reported. Li et al. demonstrated that resveratrol (150 μM) triggered autophagy in human glioma cells (U251), but autophagy suppressed the resveratrol-induced apoptosis, as shown by employing autophagy inhibitors [288]. It has also been reported that resveratrol (30 μM) induced autophagy in U87 glioblastoma cells, as shown by the formation of autophagosomes and the increase in LC3 II, ATG5 and Beclin 1. The inhibition of resveratrol-induced autophagy triggered apoptosis, thus suggesting that autophagy behaved as a cytoprotective rather a cytostatic/cytotoxic mechanism [287].

A dual effect of resveratrol on autophagy was also reported for ovarian cancer cells and NSCLC cells. Lang et al. demonstrated that resveratrol-induced cell death in human ovarian cancer cells (OVCAR-3 and Caov-3; 30 μM) was mediated by both apoptosis and autophagy. Indeed, it has been shown that the selective inhibition of autophagy with chloroquine or with a siRNA that blocks the expression of ATG5, attenuates resveratrol-induced apoptotic cell death [289]. Zhong et al. confirmed these effects of resveratrol in the same cell lines [291]. Moreover, it has been reported that resveratrol inhibited the cell migration of OVCAR-3 cells by inhibiting the Akt/mTOR pathway and STAT3 and by inducing autophagy. In particular, resveratrol promoted the synthesis and accumulation of Ras homolog member I (ARH-I), a tumor suppressor that positively regulates autophagy and that effectively inhibits cell migration in ovarian cancer cells [341]. Tan et al. demonstrated that resveratrol reduced tumor growth and induced autophagy in an in vivo ovarian cancer model. Nude mice were injected with A2780 ovarian carcinoma cells and treated with resveratrol (160 mg/kg/day). Tumors from mice treated with resveratrol showed autophagosomes, consistent with the induction of autophagy [292]. Conversely, resveratrol (25 µM) induced autophagy, which inhibited apoptosis in SKOV-3 human ovarian cancer cells. When an autophagy inhibitor was simultaneously applied, resveratrol efficiently promoted apoptosis, suggesting that autophagy protects cells from resveratrol-induced apoptosis [290]. Similarly, resveratrol (20 μM) suppressed the proliferation of Ishikawa endometrial carcinoma cells by activating AMPK and ERK signaling and by inducing autophagy and apoptosis. However, the combined treatment with resveratrol and autophagy inhibitors resulted in greater cytotoxicity in Ishikawa cells, compared with resveratrol treatment alone [293]. It has also been demonstrated that resveratrol, by acting as a protein (caloric) restriction mimetic, induces autophagy as a cell survival mechanism under nutrient shortage in OVCAR-3 ovarian cancer cells, which is more efficient than starvation [341,342].

Resveratrol induced cytoprotective autophagy as well as cell death-mediated autophagy in NSCLC cells. Zhang et al. investigated the relationship between apoptosis and autophagy in resveratrol-treated NSCLC A549 cells. The results showed that resveratrol (50 μM) increased autophagy and autophagy-mediated degradation of p62. Immunocytochemistry revealed that p62 co-localized with Fas/Cav-1 complexes, which are known to induce apoptosis, through caspase-8 activation and the cleavage of Beclin 1. The inhibition of autophagy by siRNA, which mediated the repression of Beclin 1, also blocked resveratrol-induced apoptosis, showing that apoptosis activation was dependent on autophagy [294]. Conversely, it has been shown that resveratrol (200 μM) induced cytoprotective autophagy by upregulating SIRT1 expression, by activating p38 MAPK and by inhibiting the Akt/mTOR pathway in A549 and H1299 cells. The inhibition of autophagy enhanced the anticancer effect of resveratrol by promoting apoptosis [295]. Kumar et al. demonstrated that resveratrol (50 μM) mediated its anticancer effects in H1299 and MCF-7 cells by downregulating the TP53-induced glycolysis and apoptosis regulator (TIGAR), which resulted in ROS-mediated induction of cell death by apoptosis and by autophagy for cell survival. Indeed, they also showed that treatment with chloroquine synergized with resveratrol to promote cell death by blocking the protective autophagy induced by TIGAR downregulation [343]. Other studies reported that resveratrol synergized with an anticancer drug in the treatment of NSCLC. Resveratrol synergized with gefitinib to suppress the growth of gefitinib-resistant PC9/G NSCLC cells by inhibiting EGFR activation. In addition, resveratrol (40 μM) enhanced gefitinib-induced apoptosis, autophagy, G2/M phase cell cycle arrest and senescence. The inhibition of autophagy led to a reduction in cell viability accompanied by high levels of apoptosis and by a reduction in cell senescence, suggesting that autophagy antagonized apoptosis and triggered senescence during co-treatment [296]. Furthermore, resveratrol (2.5 μM) combined with cisplatin synergistically induced apoptosis by triggering autophagic cell death through the downregulation of the Akt pathway in A549 cells. In this way, resveratrol enhanced cisplatin’s cytotoxic effects in NSCLC [297]. Conversely, Lee et al. reported that resveratrol (30 μM) and cisplatin induced apoptosis through oxidative mitochondrial damage, and autophagy in malignant mesothelioma cells (MSTO-211H and H-2452). However, the inhibition of autophagy with bafilomycin A1 made cells more sensitive to cisplatin/resveratrol-induced cytotoxicity, suggesting that the activation of autophagy, as an adapted survival mechanism, may be involved in the chemoresistant phenotype of these cells [298].

The induction of a cytoprotective autophagy by resveratrol was also reported in esophageal carcinoma, melanoma, colon cancer and breast cancer. Resveratrol inhibited the growth of squamous esophageal carcinoma cells (EC109 and EC9706) by inducing apoptosis. Resveratrol also induced AMPK/mTOR pathway-independent autophagy in these cells. However, the use of pharmacological inhibitors of autophagy demonstrated that resveratrol-induced autophagy conferred the protection of ESCC cells against apoptosis, and the blockade of autophagy enhanced the apoptosis associated with resveratrol treatment [299]. Resveratrol also inhibited the proliferation of B16 melanoma cells through the induction of apoptosis. In addition, this induced protective autophagy in cells, thus suggesting that the inhibition of autophagy could also be a strategy to improve the efficacy of resveratrol for the treatment of this type of cancer [300]. Moreover, the inhibition of autophagy enhanced resveratrol-induced caspase activation and apoptosis in breast and colon cancer cells (HCT-116 and MDA-MB-231; 60–120 µM) [344]. Conversely, it has been reported that resveratrol (64 µM) induced caspase-dependent and -independent cell death in breast cancer cells sensitive to caspase-3-dependent apoptosis (MCF-7^casp-3^) and induced caspase-independent cell death in breast cancer cells unresponsive to caspase-3-dependent apoptosis (MCF-7^vc^). Resveratrol also induced Beclin 1-independent autophagy in both cell lines [301]. Moreover, resveratrol (50 and 100 µM) induced autophagy by directly inhibiting the mTOR/ULK1 pathway in MCF-7 cells. Resveratrol inhibited mTOR by competing with ATP [345]. Hyperactivation of mTORC1 is frequent in breast cancer and it has been reported that resveratrol can act in combination with the mTORC1 inhibitor rapamycin to induce apoptosis of breast cancer cells. While treatment with rapamycin activated Akt, the combined treatment of rapamycin with resveratrol (100 µM) blocked the activation of Akt signaling in MCF-7 and MDA-MB-231 cells. Moreover, resveratrol suppressed rapamycin-induced autophagy, decreasing LC3 II accumulation and restoring p62 levels to above baseline, and led to cell death by apoptosis [302]. Rai et al. also demonstrated that resveratrol enhanced the anticancer cytotoxic effects of doxorubicin and salinomycin in the same breast cancer cells, by a similar mechanism of action. Indeed, the combined treatment downregulated Akt, decreased LC3B and Beclin 1 protein expression, reduced the proteins associated with inflammation, inhibited epithelial–mesenchymal transition and caused apoptosis [303,304].

Back et al. reported that resveratrol (50 μM) induced premature senescence in A431 human epidermoid carcinoma cells by attenuating autophagy through the blockade of autolysosome formation, as shown by the absence of colocalization of LC3 and LAMP-2. Moreover, resveratrol inhibited mTORC2 activity, by downregulating Rictor protein [305].

Other studies investigated the effects of a natural dimethylated analog of resveratrol with greater bioavailability, pterostilbene (trans-3,5-dimethoxy-4′-hydroxystilbene), which is primarily found the wood of a tree, *Pterocarpus marsupium*, and is a traditional herbal medicine used for the treatment of diabetes [346]. In multiple research findings, pterostilbene was shown to be an effective apoptotic and autophagic agent, able to inhibit cancer cell viability, to induce cell cycle arrest, to alter genes involved in apoptosis, to promote autophagy-related proteins, and to inhibit metastasis [347]. Pterostilbene (43 μM) induced autophagy and apoptosis in HL-60 human leukemia cells. Indeed, it induced intensive cytoplasmic vacuolation and an accumulation of vacuolar structures, suggesting that the treatment resulted in reduced autophagic degradation, which, in turn, led to cell death [306]. Wang et al. reported that pterostilbene (50 µM) inhibited cell growth, and induced cell cycle arrest and apoptosis in breast cancer cells (Bcap-37 and MCF-7), by inhibiting Wnt signaling. In addition, it induced autophagy in these cells. However, the blockade of autophagy enhanced the pterostilbene-cytotoxic effects, suggesting that autophagy played a cytoprotective role in these cells [307]. Conversely, it has been shown that pterostilbene (50 and 70 μM) induced intrinsic apoptosis, as well as autophagic cell death in cisplatin-resistant human oral cancer (CAR) cells, as shown by the increase in the protein expression of ATG5, ATG7, ATG12, Beclin 1 and LC3 II and by the formation of AVOs. In addition, it inhibited MDR1 expression and the phosphorylation of Akt [308]. Similarly, α-Viniferin (10–100 µM), an oligostilbene of trimeric resveratrol, was demonstrated to be able to activate apoptosis through AMPK-mediated activation of autophagy in human prostate cancer cells (DU145 and PC-3). The results showed the upregulation of apoptosis and autophagy-associated proteins (ATG5, ATG7, ATG12, and LC3A), the activation of AMPK and a decrease in the glucocorticoid receptor expression upon treatment [348].

#### 3.2.6. Xanthones

Different dietary compounds of the xanthone class have demonstrated to modulate autophagy. α-Mangostin (5-hydroxy-2-methyl-1,4-naphthoquinone; 2.5–10 µM) also exerted anticancer effects in human glioblastoma cells (GBM8401 and DBTRG-05MG) through the induction of autophagic cell death, but not apoptosis, in transplanted glioblastomas in nude mice (2 mg/kg/day, i.p., for 28 days). These effects were achieved by the activation of the AMPK pathway, which resulted in the suppression of mTORC1 activity and its downstream targets [309]. Wang et al. also reported the promotion of autophagy and apoptosis through the inhibition of the PI3K/Akt/mTOR pathway and the inhibition of inflammation by α-mangostin (5 and 20 mg/kg, i.p., daily) in mice with DMBA/12-*O*-tetradecanoylphorbol-13-acetate (TPA)-induced skin cancer. In this way, treatment with xanthone suppressed the tumor growth and reduced the incidence rate of tumors in mice [310]. Conversely, another study suggested a different effect exerted by α-mangostin in modulating autophagy in CML cells. Chen et al. demonstrated that the xanthone (5–20 µM) inhibited the proliferation of CML cells (K562, KBM5 and KBM5-T135I) by activating apoptosis and autophagy. However, the use of chloroquine showed the enhancement of cell death by apoptosis, thus suggesting that autophagy played a protective role in the cell death induced by α-mangostin in CML cells [311].

Other studies investigated the induction of autophagy by different xanthones from *Garcinia hanburyi*, gambogic acid, gambogenic acid and isogambogenic acid, in CML cells and in lung cancer cells [312,313,314,316]. The treatment of CML K562 cells with gambogic acid (0.5–2 µM) resulted in the activation of cell death by apoptosis and autophagy, with the accumulation of autophagic vacuoles, an increase in autophagy-related proteins (LC3 and Beclin 1) and a decrease in p62 protein levels. The inhibition of autophagy also inhibited apoptosis, suggesting crosstalk between the two pathways induced by gambogic acid [312]. The inhibition of cancer cell growth through the induction of autophagy by this xanthone was also demonstrated in NSCLC cells (NCI-H441). The induction of autophagy was mediated by ROS generation [313]. The gambogenic acid (1.25–2.5 µM) also triggered autophagy, but not apoptosis, and reduced cell viability in lung cancer cells (H1975, H460). In particular, the induction of autophagy was mediated by the activation of GSK3β and the inactivation of Akt/mTOR pathway by gambogenic acid [316]. Similarly, isogambogenic acid (2.5–10 µM) induced only autophagic cancer cell death and not apoptosis in human NSCLC cells (A549 and H460) and in a xenograft model (20 mg/kg, i.v., every 2 days, for 24 days), through the inhibition of the Akt/mTOR pathway. The results showed the formation of autophagic vacuoles, an increase in LC3 conversion, a decrease in p62 and an increase in autophagy-related proteins Beclin 1, ATG7 and ATG5-ATG12 complex [314]. Another study demonstrated that gambogenic acid (1.5–12 µM) induced autophagy in A549 and HeLa cells and in a xenograft tumor model (16 mg/kg, i.v., twice a week for 3 weeks) as shown by the formation of vacuoles, the increase in LC3 II, the activation of Beclin 1 and the decreased phosphorylation of p70S6K, thus indicating the inhibition of the mTOR activity. However, autophagic flux was blocked with the inhibition of the degradation of p62 and the acidification of vacuoles, which led to the suppression of the fusion between autophagosomes and lysosomes. The blocking of autophagic flux played a pro-death role in cells by activating apoptosis [317]. Similarly, another study reported that treatment of esophageal cancer cells (TE13) with gambogic acid (0.25–1 µM) increased their radiosensitivity, through the induction of apoptosis and autophagy mediated by ROS generation and the inhibition of the Akt/mTOR pathway. However, autophagic flux was blocked, thus suggesting that gambogic acid co-treatment resulted in interference in the circulation of materials, which is harmful to cancer cells [349]. Conversely, Zhang et al. demonstrated that gambogic acid (0.25–1.5 µM) induced cytoprotective autophagy mediated by ROS and the inhibition of Akt/mTOR signaling in colon cancer cells (HCT-116, SW260) and in a colon cancer xenograft model (8 mg/kg, i.p., daily). They showed that gambogic acid triggered autophagy in these cells, but the inhibition of this process resulted in the enhancement of cell death and apoptosis induced by xanthone [315].

The induction of cytoprotective autophagy, through JNK activation, was also reported for a 4-prenylated xanthone isolated from mangosteen, gartanin (10–40 µM), in HCC (Hep3B, HepG2, Huh7 cells), as indicated by the presence of acridine orange staining of intracellular AVOs, the conversion of LC3 I to LC3 II, a decrease in p62 and in LC3-positive autophagosomes and autolysosomes. However, the use of autophagy inhibitors showed the enhancement of gartanin-induced apoptotic cell death [318]. Conversely, different studies reported the induction of autophagy, as a cell death mechanism, mediated by gartanin [319,320,321]. Liu et al. demonstrated that gartanin (10–25 µM) induced both apoptosis and autophagy through the inhibition of the mTOR pathway in urinary bladder cancer cells (T24 and RT4) [319]. The suppression of the PI3K/Akt/mTOR pathway, which led to the induction of autophagy by gartanin (10 µM), was also reported in human glioma cells (T98G). In this cell line, the activation of autophagy, which also regulated cell cycle arrest, was the mechanism that mediated the anti-proliferative effects of gartanin [320]. Similarly, it was demonstrated that gartanin (6–24 µM) inhibited the growth of prostate cancer cells (22Rv1, PC-3) by triggering autophagy, as shown by the increase in LC3 II punctuate staining and the increase in LC3 II expression [321].

Other xanthones induced autophagy in different cancer cells as well. For example, formoxanthone C (20 µg/mL), isolated from *Cratoxylum formosum* ssp. *pruniflorum*, reversed the etoposide resistance by inducing both apoptosis and autophagy in MDR human A549 lung cancer cells (A549RT-eto) [322]. Similarly, mangiferin (5–20 µM), from *Mangifera indica*, inhibited cell growth by activating apoptosis and autophagy in gemcitabine-resistant pancreatic carcinoma cells (Mia-PaCa2) [323]. A xanthone-rich extract from *Gentiana dinarica*-transformed roots (50 µg/mL), and its main active component, norswertianin (40 µM), reduced the growth of glioblastoma cells (U251) and stimulated autophagy (via an increase in intracellular acidification, the conversion of LC3 I to LC3 II, and decreased levels of p62), through the inhibition of the Akt/mTOR pathway [324]. The induction of autophagy was also reported for xanthone V_1_ (10–20 µM) derived from the leaves of *Garcinia cowa* in HeLa cells [325]. Conversely, Yu et al. reported that cudraxanthone D (50 µM), derived from the root bark of *Cudrania tricuspidata*, decreased proliferation and inhibited the metastatic potential of the cells through the attenuation of autophagy (decrease in autophagic vacuoles) in human OSCC (Ca9–22 and SCC25) cells. Thus, this xanthone acted as an autophagy inhibitor [326].

## 4. Conclusions

Polyphenols are a large class of compounds of plant origin present in our diet, with multiple beneficial effects on human health, which are able to modulate inflammation, the immune system, and multiple signaling pathways involved in carcinogenesis. One of the hallmarks of cellular transformation is the alteration of cell death mechanisms, including apoptosis, autophagy and necrosis. The role of autophagy in cancer appears to be controversial: autophagy exerts either tumor suppression or tumor survival in cellular stress conditions. However, a dual dynamic role of autophagy is more often observed. The tumor type and stage and the genetic context and duration of treatments might influence tumor response through the activation or repression of the autophagic process. Accordingly, a definite and unique impact of autophagy cannot be inferred [350]. In addition, each study should follow universal guidelines for autophagy determination. Thus, autophagy modulation could represent an attractive therapeutic strategy for cancer.

In this regard, polyphenols possess the ability to modulate autophagy, as shown by the wide range of both in vitro and in vivo studies reported in this review. Polyphenols can induce autophagy to trigger cancer cell death and, in this case, autophagy cooperates with apoptosis to inhibit tumor growth. Moreover, autophagy can influence the stromal compartment of tumors, containing cancer-associated fibroblasts, adipocytes and immune cells. Both the drug uptake and the immune infiltration at the tumor site can be influenced by the tumor microenvironment, which can counteract immunotherapy and contribute to a worse prognosis. The crosstalk between the tumor and stroma supports the proliferation and metabolism of the tumor itself and autophagy has a role in this dialogue. The increased cellular stress activates autophagy in cancer-associated fibroblasts as a pro-survival mechanism, which promotes tumor growth, while the decreased autophagy in the fibroblasts counteracts tumor progression [351,352]. In a model of cholangiocarcinoma, resveratrol interfered with the release of cytokines by cancer-associated fibroblasts, which activated autophagic flux in cancer cells and halted their migration [353].

Recently, it was also shown that autophagy can be targeted by epigenetic modifiers during cellular transformation. Indeed, one of the mechanisms of tumor growth is supported by the epigenetic modifications of genes regulating autophagy [354]. Several studies have highlighted the potential of polyphenols to act as epigenetic modifiers of autophagy, by inducing a change in DNA methylation, in histones and in the expression of miRNA [354,355,356,357].Thus, the use of polyphenols could be a useful tool for the treatment of cancer.

On the other hand, polyphenols can also induce cytoprotective autophagy, which acts as antagonist of apoptosis, to promote cell survival. In this case, treatments with autophagy inhibitors combined with polyphenols, or the use of those few polyphenols that directly inhibit autophagy, could be a novel promising strategy to enhance anticancer activities.

It is worth noting that the high or low concentrations at which the polyphenol is used could induce different effects, pro- or anti-autophagic, and this issue is an important aspect to consider when using polyphenols in combination with approved chemotherapies. Chemo- and/or radio-resistance to cancer therapies could also develop secondarily to cytoprotective autophagic mechanisms activated by cancer cells. Several studies have demonstrated the role of polyphenols in enhancing the cytotoxic effects of anticancer drugs by inhibiting drug-induced autophagy.

One of the main drawbacks in the use of polyphenols for patient treatments, also in combination with conventional chemotherapies, is their low bioavailability in the human body, which affects the effective dose delivered to cancer cells. Thus, the development of novel formulations (nanosuspensions, solid lipid nanoparticles, liposomes, gold nanoparticles, polymeric nanoparticles) with a better bioavailability, activity, stability and pharmacokinetics is underway in order to improve the efficacy of these compounds [358]. The use of these novel formulations facilitates the intracellular uptake of the polyphenol and reduces its toxicity and clearance rate [359]. Accordingly, clinical trials evaluating the efficacy of polyphenols, novel formulations, and their combination with conventional therapies are ongoing for the treatment of patients with different types of cancer [360,361,362,363,364,365,366].

Although polyphenols represent a novel therapeutic strategy for the treatment of cancer, more detailed investigations would be useful and are necessary to clarify their role as autophagy-activator or autophagy-inhibitor compounds.

## Figures and Tables

**Table 1:** In vitro and in vivo effects of polyphenols on autophagy in cancer.

Polyphenol	In Vitro Model	In Vivo Model	Effects on Autophagy	Reference
**Flavonoids**				
***Flavonols***				
Quercetin	MCF-7 breast cancer cells and HeLa cervical cancer cells (90 µM)		Induction of autophagy ↑ Autophagosomes and autolysosomes ↓ mTOR pathway	[58]
	Burkitt lymphoma cells (100 µM)		Induction of autophagy ↑ LC3 II expression ↓ PI3K/Akt/mTOR pathway	[59]
	MCF-7 and MDA-MB-231 breast cancer cells (30 µM)	BALB/c nude mice s.c. injected with MCF-7 cells (50 mg/kg, i.p., twice daily)	Induction of autophagy ↑ LC3 II/I ratio ↑ LC3-puncta structures ↓ Akt/mTOR pathway	[60]
	A549 NSCLC cells (20–80 µM + TRAIL)		Induction of autophagy ↑ LC3 II expression ↑ Autophagosomes ↓ Akt/mTOR pathway ↑ TRAIL-induced cell death	[61]
		NOD/SCID mice s.c. injected with HL-60 AML cells (Quercetin 120 mg/kg, i.p., every 4 days; green tea: 100 mg/kg, p.o., daily)	Induction of autophagy ↑ Beclin 1, ATG7, ATG5-ATG12 expression ↑ LC3 II positive cells	[62]
	MOGGCCM anaplastic astrocytoma and T98G glioblastoma multiforme (30–50 µM + Sorafenib)		Induction of autophagy ↑ AVOs ↑ Beclin 1 and LC3 II expression	[63]
	U373MG malignant glioma cells (25–100 µM)		Induction of cytoprotective autophagy ↑ AVOs ↑ LC3 I to LC3 II conversion	[64]
	U87 and U251 glioma cells (25–100 µM)	Sprague Dawley rats intracranially injected with rat glioma C6 cells (100 mg/kg, i.v., daily)	Induction of cytoprotective autophagy ↑ Autophagosomes ↑ LC3 II expression	[65]
	AGS and MKN28 gastric cancer cells (AGS: 10–40 µM; MKN28: 40–160 µM)	BALB/c mice s.c. injected with MKN28 cells (50 mg/kg, i.p., daily)	Induction of cytoprotective autophagy ↑ Autophagosomes ↑ AVOs ↑ LC3 I to LC3 II conversion ↑ Beclin 1, ATG7, ATG5-ATG12 expression ↓ Akt/mTOR pathway	[66]
	BC3 and BCBL1 primary effusion lymphoma cells (50 µM)		Induction of cytoprotective autophagy ↑ LC3-puncta structures ↑ LC3 II expression ↓ PI3K/Akt/mTOR pathway	[67]
	CAOV-3 and primary P#1 ovarian cancer cells (40–80 µM)	NOD/SCID mice i.p. injected with CAOV-3 cells (80 mg/kg, i.p., twice a week)	Induction of cytoprotective autophagy ↑ Autophagosomes ↑ LC3-puncta structures ↑ LC3 I to LC3 II conversion ↑ Beclin 1 and ATG5 expression	[68]
	P39 leukemia cells (50 µM)		Induction of cytoprotective autophagy	[69]
	HL-60 AML cells (100 µM)		Induction of cytoprotective autophagy	[70]
	HeLa cervical cancer cells (50 µM)		Induction of cytoprotective autophagy	[71]
CHNQ	HCT-116 and HT-29 colon cancer cells (HCT-116: 25 µM) (HT-29: 40 µM)		Induction of autophagy ↑ LC3 I to LC3 II conversion ↑ LC3-puncta structures ↑ AVOs ↓ Akt phosphorylation	[72]
8-CEPQ	SW620 and HCT-116 colon cancer cells (15 µM)		Induction of autophagy ↑ AVOs ↑ LC3-puncta structures ↑ LC3 II, Beclin 1 and ATG7 expression ↓ p62 expression ↑ ERK pathway	[73]
GQ	A549 and NCI-H1975 NSCLC cells (25–35 µM)		Induction of autophagy ↑ Autophagosomes ↑ LC3 II and Beclin 1 expression ↓ p62 expression	[74]
Hyperoside	A549 NSCLC cells (0.5–2 mM)		Induction of autophagy ↑ LC3 II expression ↑ Autophagosomes ↓ p62 expression ↓ Akt/mTOR/p70S6K pathway ↑ ERK pathway	[75]
	SKOV-3 and HO-8910 ovarian cancer cells (50–100 µM)		Induction of autophagy ↑ MDC-labelled vacuoles ↑ LC3 II expression	[76]
Isorhamnetin	A549 NSCLC cells (2–8 µM)	BALB/c nu/nu mice s.c. injected with A549 cells (0.5 mg/kg, i.p., daily)	Induction of cytoprotective autophagy ↑ LC3 II and Beclin 1 expression ↑ LC3-puncta structures ↑ MDC-labelled vacuoles	[77]
Rutin	D54MG glioblastoma multiforme cells (50–200 µM + TMZ)	BALB/c athymic mice s.c. injected with U87 glioma cells or intracranially injected with U87 cells (20 mg/kg, i.p., daily)	Inhibition TMZ-induced autophagy ↓ LC3 II expression ↓ JNK activity ↑ Drug-induced cytotoxicity	[78]
Taxifolin	HeLa cervical cancer cells (100 µM + Andrographolide)		Inhibition andrographolide-induced autophagy ↓ LC3-puncta structures ↓ AVOs ↑ Drug-induced cytotoxicity	[79]
Quercetin nanoparticles (NPs)	U87 glioma cells (30–40 µg/mL)	BALB/c nu/nu nude mice s.c. injected with U87 cells (40 and 80 mg/kg, i.p., daily)	Induction of autophagy ↑ LC3 I to LC3 II conversion ↑ Beclin 1 expression ↓ p62 expression ↓ Akt/mTOR pathway	[80]
	Caski cervical cancer cells (10–20 µg/mL)	BALB/c nu/nu nude mice s.c. injected with Caski cells treated with NPs	Induction of autophagy ↑ Autophagosomes ↓ Akt/mTOR pathway	[81]
Kaempferol	SK-Hep-1 HCC cells (50–100 µM)		Induction of autophagy ↑ Autophagosomes ↑ AVOs ↑ LC3-puncta structures ↑ LC3 II, ATG5, ATG7, ATG12 and Beclin 1 expression ↓ Akt/mTOR pathway	[82]
	A549 NSCLC cells (20–50 µM)		Induction of autophagy ↑ LC3 II/I ratio ↑ ATG7 and Beclin 1 expression ↓ p62 expression ↑ LC3-puncta structures ↓ PI3K/Akt pathway	[83]
	SNU-216 gastric cancer cells (50 µM)		Induction of autophagy ↑ LC3 II/I ratio ↑ ATG7 and Beclin 1 expression ↓ p62 expression	[84]
	AGS and SNU-638 gastric cancer cells (50 µM)		Induction of autophagy ↑ LC3 I to LC3 II conversion ↑ LC3 II, Beclin 1 and ATG5 expression ↓ p62 expression ↑ LC3-puncta structures	[85]
Kaempferol or CAPE	RKO and HCT-116 colon cancer cells (RKO: CAPE 36.87 µM, kaempferol 17.42 µM) (HCT-116: CAPE 3.326 µM, kaempferol 9.427 µM)		Induction of autophagy ↑ MDC-labelled vacuoles	[86]
Juglanin	MCF-7 and SK-BR-3 breast cancer cells (2.5–10 µM)	BALB/c-nude mice s.c. injected with MCF-7 cells (5 and 10 mg/kg, i.p., daily)	Induction of autophagy ↑ Autophagosomes ↑ LC3 II expression	[87]
Kazinol A	T24 and cisplatin-resistant T24R2 bladder cancer cells (20 µM)		Induction of autophagy ↑ LC3-puncta structures ↑ LC3 I to LC3 II conversion ↓ mTOR phosphorylation	[88]
Dihydromyricetin	A431 epidermoid carcinoma cells (25–100 µM)		Induction of autophagy ↑ Beclin 1 and LC3 II expression	[89]
	HepG2 HCC cells (10–50 µM)		Induction of autophagy ↑ Beclin 1 and LC3 II expression	[90]
	SK-MEL-28 human melanoma cells (100 μM)		Induction of cytoprotective autophagy ↑ Beclin 1 and LC3 II expression ↓ p62 expression Activation NF-κB pathway	[91]
	CAL-27 OSCC cells (50 μM)		Induction of cytoprotective autophagy ↑ Beclin 1 and LC3 II expression ↓ p62 expression	[92]
***Flavan-3-ols***				
HLP extract and ECG	A375 melanoma cells (HLP: 100–250 μg/mL; ECG: 100 µM)		Induction of autophagy ↑ AVOs ↑ LC3 II, Beclin 1, ATG9, ATG16, ATG5-ATG12 expression ↓ Akt/mTOR pathway	[93]
EGCG	SSC-4 OSCC cells (20 µM)		Induction of autophagy ↑ MDC-labelled vacuoles	[94]
	4T1 breast cancer cells (10, 20, 40 µM)	BALB/c mice s.c. injected with 4T1 cells (5, 10, 20 mg/kg, i.p., daily)	Induction of autophagy ↑ LC3 II/I ratio, Beclin 1, ATG5 expression ↑ LC3-puncta structures ↑ Autophagosomes	[95]
	HepG2 HCC cells (25–50 µM)		Induction of autophagy ↑ MDC-labelled vacuoles ↑ LC3-puncta structures ↑ LC3 II expression Degradation of AFP aggregates	[96]
	HCT-116 colorectal cancer cells (12.5 µM + 2 Gy radiation)		Induction of autophagy ↑ LC3 II mRNA expression ↑ Sensitivity to radiation	[97]
	HepG2 HCC cells PANC-1 pancreatic cancer cells (20 µM + PEF + US)		Induction of autophagy ↑ MDC-labelled vacuoles ↑ LC3 II expression ↓ Akt phosphorylation	[98]
	GBM15, GBM16 primary glioblastoma cells (500 µM)		Induction of autophagy ↑ MDC-labelled vacuoles ↑ LC3 II expression	[99]
	EHMES-10, ACC-meso and Y-meso malignant mesothelioma cells (50–250 µM)		Induction of cytoprotective autophagy ↑ LC3 II expression	[100]
JP8	B16-F10 melanoma cells (20 µM)	C57BL/6 mice s.c. injected with B16-F10 cells (25 and 50 mg/kg, i.p., daily)	Induction of autophagy ↑ LC3 I to LC3 II conversion ↑ LC3-puncta structures ↓ p62 expression	[101]
Green tea extract	A549 NSCLC cells (25, 50, 150 µM)		Induction of cytoprotective autophagy ↑ Autophagosomes and autolysosomes ↑ AVOs ↑ LC3-puncta structures	[102]
Polyphenon E^®^	PNT1a prostate cancer cells (35 µg/mL)		Induction of cytoprotective autophagy ↑ LC3-puncta structures ↑ LC3 I to LC3 II conversion	[103]
Tea polyphenols + anticancer drugs	T24 cells and BIU87 bladder cancer cells (100 µM + Epirubicin)		Inhibition epirubicin-induced autophagy ↓ LC3 II induction ↓ Autophagosomes and autolysosomes ↑ Sensitivity to epirubicin	[104]
	PC-3 and DU145 castration-resistant prostate cancer cells (20 µM + Docetaxel)		Inhibition docetaxel-induced autophagy ↓ LC3 II expression ↑ mTOR activation ↑ Sensitivity to docetaxel	[105]
EGCG + anticancer drugs	Hep3B hepatoma cells (10–40 µg/mL + DOX)	Nude mice s.c. injected with Hep3B cells (50 mg/kg, intragastric, daily + DOX)	Inhibition DOX-induced autophagy ↓ Autophagosomes ↓ ATG5, Beclin 1 mRNA and protein ↑ Sensitivity to DOX	[106]
	SaoS2 and U2OS cells osteosarcoma cells (20 µg/mL + DOX)		Inhibition DOX-induced autophagy ↓ LC3-puncta structures ↓ ATG5, Beclin 1 mRNA ↓ LC3 II/I ratio ↑ p62 expression ↑ Sensitivity to DOX	[107]
	A549 NSCLC cells (34 µM + Gefitinib)		Inhibition gefitinib-induced autophagy ↓ LC3-puncta structures ↓ LC3 II/I ratio, ATG5 expression ↑ p62 expression ↑ Sensitivity to gefitinib	[108]
	DLD-1 and HT-29 colon cancer cells (100 µM + Cisplatin/Oxaliplatin)		↑ Drugs-induced autophagy ↑ LC3 I to LC3 II conversion ↑ LC3 II expression ↑ Autophagosomes ↑ AVOs	[109]
	Cisplatin-resistant CAR oral cancer cells (50 µM)		Induction of autophagy ↑ AVOs ↑ Autophagosomes ↑ LC3-puncta structures ↑ ATG5, ATG7, ATG12, Beclin 1 and LC3 II expression ↓ Akt/STAT3 pathway	[110]
	PC-3 prostate cancer cells (5, 50 µM + Bortezomib)		Induction of autophagy ↑ LC3-puncta structures ↑ LC3 I to LC3 II conversion Antagonized drug-induced cytotoxic effect	[111]
	HCT-116 colorectal cancer cells (5–20 µM + TRAIL)		Induction of autophagy ↓ p62 expression and accumulation Antagonized drug-induced cytotoxic effect	[112]
***Flavones***				
Isocannflavin B	Estrogens sensitive ER^+^ T47-D and insensitive ER^−^ MDA-MB-231 cells (25 µM)		Induction of autophagy in ER^+^ cells	[113]
Apigenin	Primary epidermal keratinocytes and COLO16 cutaneous squamous cell carcinoma cells (20–80 µM + UVB)		Restoring of autophagy ↑ LC3 II	[114]
	TF1 erythroid subtype leukemia cells (100 µM)		Induction of autophagy ↑ ATG5, ATG12 expression	[115]
	MM-F1, MM-B1 and H-Meso-1 malignant mesothelioma cells (50 μM)		No effects on autophagy	[116]
	HT-29 colon cancer cells (15–60 µM)		Induction of autophagy ↑ Autophagosomes ↑ Beclin 1 and LC3 II expression ↓ p62 expression	[117]
	BCPAP papillary thyroid carcinoma cells (12.5–50 µM)		Induction of autophagy ↑ Beclin 1 and LC3 II expression ↓ p62 expression	[118]
	HepG2 HCC cells (10–40 µM)		Induction of cytoprotective autophagy ↑ LC3 II, ATG5 and Beclin 1 expression ↓ PI3K/Akt/mTOR pathway	[119]
	HCT-116 colon cancer cells (6.25–50 µM)		Induction of cytoprotective autophagy ↑ Beclin 1 and LC3 II expression ↓ p62 expression	[120]
Wogonin	NPC-TW076 and NPC-TW039 nasopharyngeal carcinoma cells (50 µM)		Induction of cytoprotective autophagy ↑ LC3 II expression ↑ Autophagosomes and autolysosomes ↓ Akt/cRaf/ERK pathway	[121]
	SW48 colorectal cancer cells (4–16 µM)		Induction of autophagy ↑ Beclin 1 and LC3 II	[122]
	BGC-823 gastric cancer cells (10, 50 and 200 µM + Oxaliplatin)		Induction of autophagy ↑ LC3 II	[123]
Baicalein	SMMC-7721 and Bel-7402 HCC cells (100 and 200 µM)		Induction of cytoprotective autophagy ↑ LC3 II	[124]
	HepG2 HCC cells (12.5–50 µM)		Induction of cytoprotective autophagy ↓ Akt/mTOR pathway	[125]
	HEY and A2780 ovarian cancer cells (12.5–50 µM)		Induction of cytoprotective autophagy ↑ LC3 II expression and AVOs	[126]
	CAL-27 OSCC cells (25–100 µM)		Induction of cytoprotective autophagy ↑ LC3 II, Beclin 1 and p62	[127]
	U251MG glioma cells (10–80 µM)		Induction of autophagy ↑ LC3 II expression ↑ AMPK pathway	[128]
	Follicular undifferentiated thyroid cancer cells (10–80 µM)		Induction of autophagy ↑ p62, Beclin 1, ATG5, ATG12 expression	[129]
	PC-3, MDA-MB-231 and DU145 cancer cells (5 µg/mL)		Induction of autophagy ↑ Autophagosomes ↑ AMPKα and ULK1 ↓ mTOR and Raptor	[130]
	MCF-7 and MDA-MB-231 breast cancer cells (10, 20, 40 µM)	BALB/c-nude mice s.c. injected with MCF-7 or MDA-MB-231 cells (100 mg/kg, p.o., daily)	Induction of autophagy ↑ LC3 II and Beclin 1 expression ↓ PI3K/Akt pathway	[131]
	Stem cell-like cells (TICs) isolated from mouse and human liver tumors (30 µM)		Inhibition of autophagy ↓ Autophagosomes formation ↓ GTP binding of SAR1B GTPase	[132]
Polymethoxyflavone 5-demethylnobiletin (5-DMN)	CL1-5 and A549 NSCLC cells (12.5 μM)		Induction of cytoprotective autophagy ↑ Beclin 1 expression	[133]
Luteolin	MET4 cells derived from a primary cutaneous invasive squamous cell carcinoma (50 µM)		Induction of cytoprotective autophagy ↑ Autophagosomes ↓ p62 expression	[134]
	NCI-H460 NSCLC cells (200 µM)		Induction of autophagy ↑ LC3B II expression	[135]
	Huh7 HCC cells (20 µM)		Induction of autophagy ↑ LC3 II expression ↓ p62 expression	[136]
	SMMC-7721 HCC cells (25–100 µM)		Induction of autophagy ↑ Autophagosomes ↑ LC3B II and Beclin 1 expression	[137]
Luteolin and Silibinin	U87MG and T98G glioma cells (Luteolin: 20 µM; Silibinin: 50 µM)		Inhibition of autophagy ↓ Beclin 1, LC3B I and II expression	[138]
Salvigenin	SH-SY5Y neuroblastoma cells (25–50 µM)		Induction of cytoprotective autophagy ↑ LC3 II/I ratio ↑ ATG7 and ATG12 expression	[139]
Baicalin	SMMC-7721 HCC cells (40–160 µM)		Induction of autophagy ↑ Beclin 1 expression	[140]
	Human bladder cancer T24 cells (100–200 µM)		Induction of autophagy ↓ p-Akt (Ser473) protein level and Akt kinase activity ↑ ATG complex, LC-3 and Beclin 1 expression	[141]
Diosmin	MCF-7, MDA-MB-231 and SK-BR-3 breast cancer cells (5–20 μM)		Induction of cytostatic and cytotoxic autophagy ↑ Oxidative stress and DNA damage	[142]
Seed extract from *Euterpe oleracea* Mart.	MCF-7 breast cancer cells (10, 20 and 40 μg/mL)		Induction of autophagy ↑ LC3B II expression	[143]
Delicaflavone	A549 and PC-9 NSCLC cells (40 μg/mL)		Induction of autophagy ↑ Autophagosomes ↑ LC3 II/I ratio ↓ p-Akt, p-mTOR and p-p70S6K	[144]
Luteoloside	A549 and H292 NSCLC cells (60 µM)		Induction of autophagy ↓ Akt/mTOR/p70S6K signaling pathway ↑ Beclin 1 and LC3 II expression ↓ p62 expression	[145]
Glychionide-A	PANC-1 pancreatic cancer cells (7–28 µM)		Induction of autophagy ↑ Beclin 1 and LC3 II expression ↓ p62 expression	[146]
Isoorientin	HepG2 HCC cells (20–80 μM)		Induction of autophagy ↑ Beclin 1 and LC3 II expression	[147]
Isovitexin	HepG2 and SK-Hep-1 HCC cells (12.5–50 μg/mL)		Induction of autophagy ↑ Beclin 1, LC3B II, ATG3 and ATG5 expression	[148]
Wogonoside	U251MG and U87MG glioma cells (250–300 µM)		Induction of autophagy ↑ AVOs ↑ LC3 II expression ↓ p62 expression ↑ p38 MAPK ↓ PI3K/Akt/mTOR/p70S6K pathways	[149]
Nobiletin	CAOV-3 and ES-2 ovarian cancer cells (40 μM)		Induction of autophagy ↑ p62 expression	[150]
	SNU-16 gastric cancer cells (12.5–50 μM)		Induction of autophagy ↑ LC3 II/I ratio ↓ p62 expression	[151]
Zapotin	HeLaPKCeA/E cancer cells with overexpressed constitutively active protein kinase C epsilon (30 μM)		Inhibition of autophagy ↓ Autophagosomes formation ↓ LC3 expression	[152]
Vitexin	SK-Hep-1 and Hepa1-6 HCC cells (100 µM)		Inhibition of cytoprotective autophagy ↓ LC3 II expression	[153]
***Anthocyanins***				
Delphinidin	MDA-MB-453 and BT474 HER-2^+^ breast cancer cells (MDA-MB-453: 80 µM) (BT474: 140 µM)		Induction of autophagy ↑ Autophagic vacuoles ↑ LC3 II expression ↑ ATG5-ATG12 expression ↓ p-Akt, p-mTOR, p70S6K, eIF4E ↑ LKB1, AMPK, ULK1, FOXO3a	[154]
	SMMC7721, HCCLM3 and MHCC97L HCC cells (80–150 µM)		Induction of autophagy ↑ Autophagic vacuoles ↑ LC3 II expression	[155]
	U2OS osteosarcoma cells (10–200 µM)		Induction of autophagy ↑ Autophagosomes ↑ LC3 II expression ↓ p62 expression	[156]
	HeLa cervical cancer cells (100 µM)	ATG5-deficient mouse embryonic fibroblasts (100 µM)	Induction of autophagy ↑ Autophagosomes and autolysosomes ↑ Colocalization LC3 II/LAMP-1 Ex vivo ↓ Autophagy	[157]
Pelargonidin	U2OS osteosarcoma cells (15–30 µM)		Induction of autophagy ↑ Beclin 1 and LC3 II expression ↓ LC3 I expression ↓ p-PI3K, p-Akt ↑ ROS	[158]
Cyanidin	786-O and ACHN RCC (25–100 µM)		↓ ATG4, p62 expression ↓ LC3 II expression ↑ EGR1, SEPW1 expression	[159]
C3G	INS-1 rat pancreatic β cells under oxidative stress condition (H_2_O_2_ treatment) (0.5–1 µM)		Reduction in H_2_O_2_-induced autophagy ↓ LC3 II expression ↓ Autophagic vacuoles ↓ MDC-labelled vacuoles ↑ HO-1, Nrf2	[160]
Polyphenols from Mulberry water extract (MPE)	p53^+^ HepG2 and p53^−^ Hep3B HCC cells (0.25–1 mg/mL)	DEN-induced liver cancer in Wistar rat fed with normal diet + 1–2% MPE	↑ Apoptosis of p53^+^ HepG2 ↑ Autophagy of p53^−^ Hep3B, ↑ AMPK ↓ PI3K/Akt/mTOR signaling Protection from liver damage and HCC formation	[161]
	SW1736 and HTh-7 thyroid cancer cells (10 µg/mL)		Induction of autophagy ↑ LC3 II/LC3 I ratio ↑ Autophagic vesicles ↑ LC3-puncta structures ↓ Akt/mTOR signaling	[162]
	SGC-7901 gastric cancer cells		Induction of autophagy ↑ LC3 II/I ratio ↑ Beclin 1 expression	[163]
Anthocyanins, extracted from black soybean	U2OS osteosarcoma cells (100–300 µg/mL)		Induction of autophagy ↑ LC3 I to LC3 II conversion ↑ LC3-puncta structures ↑ p-ERK1/2, p-p38 MAPK, p-JNK, p-Akt ↓ p-mTOR ↑ AMPK	[164]
Anthocyanins from Pelingo apple	MCF-7 and MDA-MB-231 breast cancer cells (2.5% v/v of Pelingo juice)		Induction of autophagy ↑ LC3 II/I ratio ↑ Autophagic vacuoles ↑ p21 expression ↓ ERK1/2	[165]
Illawarra plum extract	HT-29 colonic cancer cells (100 µg/mL)		Induction of autophagy ↑ Alteration of morphology ↑ Autophagic vacuoles ↑ SIRT1 expression ↑ Nuclear buds, micronuclei, and nucleoplasmic bridges	[166]
Cinnamtannin D1	A549 and H460 NSCLC cells (125–175 μM)		Induction of autophagy ↑ LC3-puncta structures ↑ LC3 II expression ↑ ATG5 expression ↓ Akt/mTOR ↑ ERK1/2	[167]
Proanthocyanidin-rich cranberry extract	JHAD1 and OE19 esophageal adenocarcinoma cells (75 μg/mL)		Induction of autophagy ↓ Beclin 1 expression ↑ LC3 II expression ↑ Autophagic vacuoles	[168]
***Flavanones***				
Hesperidin		Male Swiss albino mice i.p. injected with AOM (25 mg/kg, p.o., daily)	Induction of autophagy ↑ Beclin 1 expression ↑ LC3 II expression ↓ PI3K/Akt/GSK-3β and mTOR pathways	[169]
Naringin		Male C57BL/6 mice i.p. injected with AOM and DSS (50 and 100 mg/kg, p.o., daily)	Inhibition ER-stress mediated autophagy Inhibition formation of autophagosomes	[170]
	AGS gastric cancer cells (2 mM)		Induction of autophagy Formation of cytoplasmic vacuoles and autophagosomes ↑ Beclin 1 and LC3 II expression ↓ PI3K/Akt/mTOR pathway ↑ MAPKs	[171]
Pinocembrin	B16-F10 and A375 melanoma cells (50–150 µM)	C57BL/6 mice s.c. implanted into the oxter with B16-F10 cells (50 mg/kg or 75 mg/kg, i.v., daily)	Inhibition of autophagy ↑ LC3 I expression ↓ ATG5 and ATG5-ATG12 expression ↓ Beclin 1 expression ↑ p62 expression ↓ AVOs ↑ PI3K/Akt/mTOR pathway	[172]
5-Methoxyflavanone	HCT-116 colon cancer cells (40 µM)		Induction of cytoprotective autophagy ↑ Autolysosomes ↑ LC3-puncta structures ↑ LC3 II expression ↑ LC3 I to LC3 II conversion	[173]
6-CEPN	SW620 and HCT-116 colon cancer cells (10 µM)		Induction of cytoprotective autophagy ↑ AVOs ↑ MDC-labelled vacuoles ↑ LC3-puncta structures ↑ LC3 II expression	[174]
Liquiritin	SGC-7901/DDP cisplatin (DDP)-resistant gastric cancer cells (80 µM + DDP)	BALB/c-nu mice s.c. injected with SGC-7901/DDP cells (15 mg/kg, i.p., daily + DDP)	Induction of autophagy ↑ Beclin 1 expression ↑ LC3 II expression ↓ p62 expression	[175]
Silibinin	DU145 prostate cancer cells (100 µM + Arsenic)		Induction of autophagy ↑ Autophagic vacuoles ↑ Beclin 1 expression	[176]
***Isoflavones***				
Genistein	A2780 ovarian cancer cells (50–100 µM)		Induction of autophagy ↑ LC3-puncta structures ↓ Akt phosphorylation	[177]
	MCF-7 breast cancer cells (100 µM)		Induction of autophagy ↑ LC3-puncta structures	[178]
		DMBA-induced mammary tumors in Sprague–Dawley rats fed with AIN93G diet supplemented with 500 ppm genistein	Inhibition of autophagy ↓ GRP78, IRE1α, ATF4 and Beclin 1 genes ↑ Sensitivity to tamoxifen	[179]
	A549 NSCLC cells (60 µM)		Induction of autophagy ↓ Bcl-xL levels ↑ LC3 II expression ↓ p62 expression Dissociation of Bcl-xL/Beclin 1 proteins	[180]
	MIA PaCa-2 pancreatic cancer cells (100 µM)	Nude mice s.c. injected with MIA PaCa-2 cells (1.3 mg, i.p., every 4 days + 5-FU)	Induction of autophagy ↓ Bcl-2 expression ↑ Beclin 1 expression ↑ AVOs ↑ 5-FU anticancer effects	[181]
	TRAIL-resistant A549 NSCLC cells (10–40 µM)		Inhibition of autophagy ↑ LC3 II expression ↑ p62 expression ↑ TRAIL-induced cell death	[182]
I3C	HT-29 colon cancer cells (40 µM + I3C 300 µM)		Induction of autophagy ↑ LC3 II expression ↓ Akt/mTOR pathway ↓ Maturation of autophagosomes	[183]
Puerarin	K562 CML cells (100 µM)		Induction of autophagy ↑ Autophagosomes ↑ LC3 II/I ratio ↑ ATG5 expression	[184]
	NCI-H441 NSCLC cells (20 µM)		Induction of autophagy ↑ MDC-labelled vacuoles ↑ ATG5 expression ↓ LC3 I expression ↓ Akt/mTOR pathway	[185]
NV-128	EOC and R182 paclitaxel- and carboplatin-resistant ovarian cancer cells (0.1–10 µM)		Induction of autophagy ↑ LC3 II expression	[186]
Furowanin A	HT-29 and SW480 colon cancer cells (2 and 5 µM)		Induction of autophagy ↑ AVOs ↑ LC3 II and Beclin 1 expression ↓ p62 expression ↑ Autophagosomes	[187]
Glabridin	Huh7 hepatoma cells (1–100 µM)		Induction of cytoprotective autophagy ↑ AVOs ↑ LC3 II and Beclin 1 expression	[188]
Celastrol	HeLa cervical cancer cells, A549 NSCLC cells, PC-3 prostate cancer cells (1.2 µM)		Induction of cytoprotective autophagy ↑ Autophagosomes ↑ LC3 II expression	[189]
	SH-SY5Y neuroblastoma cells (500 nM)		Induction of autophagy ↑ LC3 II/I ratio ↑ Autophagosomes	[190]
Phenoxodiol	KK ovarian clear cell carcinoma cells (0.5–2 µg/mL)		Inhibition of autophagy ↓ ATG7, ATG12, Beclin 1 expression ↑ Sensitivity to cisplatin	[191]
**Non-Flavonoids**				
***Coumarins***				
Hybrid of 3-benzyl coumarin seco-B-ring derivative and phenylsulfonylfuroxan	A549 NSCLC cells (50 nM)		Induction of autophagy ↑ Autophagosomes ↑ LC3 II expression ↑ LC3 I to LC3 II conversion ↑ Autophagic flux	[192]
Hybrid compound of coumarin and phenylsulfonylfuroxan	A549 and H1299 NSCLC cells (200 nM)		Induction of cytoprotective autophagy ↑ Autophagosomes ↑ LC3 I to LC3 II conversion	[193]
Feroniellin A	A549RT-eto NSCLC cells (0.05–1 mM)		Induction of autophagy ↑ LC3 I to LC3 II conversion ↑ LC3-puncta structures ↑ Beclin 1 and ATG5 expression ↓ mTOR pathway	[194]
Esculetin	HL-60 AML cells (20 µM)		Induction of autophagy ↑ Autophagosomes ↑ LC3 II and Beclin 1 expression ↓ ATG3 and p62 expression	[195]
Xanthoxyletin	SCC-1 OSCC cells (5–20 µM)		Induction of autophagy ↑ Autophagosomes ↑ Beclin 1 and LC3 II expression ↓ p62 expression	[196]
Osthole	T98G glioblastoma multiforme cells and MOGGCCM anaplastic astrocytoma cells (150–250 µM)		Induction of autophagy ↑ Autophagic cells ↑ Beclin 1 expression	[197]
Psoralen and isopsoralen	PC-3 prostate cancer cells (250 µg/mL)		Induction of autophagy ↑ AVOs ↑ LC3 II expression ↓ p62 expression	[198]
Geranylated 4-phenylcoumarin	PC-3 and DU145 prostate cancer cells (9 µM)		Induction of autophagy ↑ LC3 I to LC3 II conversion ↑ LC3-puncta structures ↑ Autophagosomes ↓ p62 expression	[199]
Hydroxypyridinone-coumarin	MHCC-97 and HepG2 HCC cells (2 µM)		Induction of autophagy ↑ ATG5, ATG3, Beclin 1 and LC3 II expression ↓ p62 expression ↑ ERK1/2 ↓ Akt	[200]
Psoralidin	HepG2 HCC cells (9–26 µM)		Induction of autophagy ↑ Autophagosomes ↑ Beclin 1 and LC3 II expression	[201]
	MCF-7 breast cancer cells (2.5–10 µM)		Induction of cytoprotective autophagy ↑ Beclin 1, LC3 II and p-ULK1 (Ser317) expression ↓ p62 expression ↓ Akt/mTOR pathway ↑ MDC-labelled vacuoles	[202]
Bergapten	MCF-7 and ZR-75 breast cancer cells (20–50 µM)		Induction of autophagy ↑ Beclin 1, PI3KII, UVRAG, AMBRA ↑ LC3 I to LC3 II conversion ↑ Autophagosomes ↑ pTEN and p38 MAPK/NF-Y pathway ↓ Akt/mTOR pathway	[203]
***Curcuminoids***				
Curcumin	A549 NSCLC cells (5–40 µM)		Induction of autophagy ↑ Autophagic vesicles ↑ MDC-labelled vacuoles ↑ Double membrane-enclosed structures ↑ LC3 II and Beclin 1 expression ↑ LC3 II/I ratio ↓ p62 expression ↓ Akt, mTOR expression	[204,205]
	A549 NSCLC cells (40 µM)		Induction of autophagy ↑ LC3-puncta structures ↑ AMPK pathway ↑ hST8Sia I, GD	[206]
	H1299 and A549 NSCLC cells (10 µM)		Induction of autophagy ↑ Beclin 1 expression ↑ LC3 II/I ratio ↑ Autophagosomes ↓ p-mTOR, p-S6, p-PI3K, p-Akt	[207]
	A549 NSCLC cells (40 µM + Galbanic acid)		Induction of autophagy ↑ LC3-puncta structures ↑ LC3 II expression ↑ Beclin 1 expression ↓ p-Akt, p-mTOR, p-p70S6K	[208]
	H157 and H1299 NSCLC cells (10 µM + Gefitinib)	BALB/c athymic nude mice s.c. injected with H157 or H1299 cells (1 g/kg, p.o., daily + Gefitinib)	Induction of autophagy ↑ LC3-puncta structures ↑ AVOs ↓ SQSTM1 In vivo ↑ LC3, Beclin 1 ↓ EGR, survivin, Sp1, HDAC1	[209]
	PANC1 and BxPC3 pancreatic cancer cells (10–80 µg/mL)		Induction of autophagy ↑ LC3 II expression ↑ Autophagosomes ↑ LC3-puncta structures	[210]
	SGC-7901 and BGC-823 gastric cancer cells (10–40 µM)		Induction of autophagy ↑ Beclin 1, ATG5, ATG3 expression ↑ LC3 I to LC3 II conversion ↓ p-mTOR, PI3K, p-Akt	[211]
	SGC-7901, BGC-823 and MKN-28 gastric cancer cells (5–20 µM)		Induction of autophagy ↑ AVOs ↑ Beclin 1, ATG7, ATG5-ATG12 expression ↑ LC3 I to LC3 II conversion ↓ p-Akt, p-mTOR, p-p70S6K	[212]
	SKOV-3 and A2780 ovarian cancer cells (SKOV-3: 10–40 µM) (A2780: 7.5–30 µM)		Induction of autophagy ↑ AVOs ↑ LC3 I/II ratio ↑ LC3-puncta structures ↑ Beclin 1, ATG3 expression ↓ p-Akt, p-mTOR, p-p70S6K, p-4EBP1	[213]
	SW620 and HCT-116 colon cancer cells (10–30 µM)		Induction of autophagy ↑ LC3 expression ↓ p62 expression ↓ YAP expression	[214]
	HCT-116 and HT-29 colon cancer cells (10–30 µM + 5-FU)	BALB/c nu/nu mice s.c. injected with HCT-116 cells (40 mg/kg, i.p., daily + 5-FU)	Induction of autophagy ↓ LC3 II/II ratio, Beclin 1 expression ↑ p62 expression ↓ p-Akt, p-mTOR, p-AMPK, p-ULK1	[215]
	HepG2 HCC cells (5–20 µM)	BALB/c nude mice s.c. injected with HepG2 cells (200 mg/kg, i.p., daily)	Induction of autophagy ↑ Beclin 1, LC3 expression ↓ Glypican-3 (GPC3) ↓ Wnt/β-catenin pathway	[216]
		Sprague Dawley rats (100–200 mg/kg, p.o., daily + TAA)	Induction of autophagy ↑ LC3 II expression ↓ ALT, AST, albumin ↑ Survival	[217]
	HeLa cervical cancer cells, HCT-116 colon carcinoma cells, HepG2 HCC cells (40 µM + JLP silencing)		Induction of cytoprotective autophagy ↑ LC3 II expression ↓ Autophagosomes-lysosome fusion and degradation with JLP silencing	[218]
	786-O and ACHN RCC cells (5–80 µM)		↑ LC3 II expression Low-dose CUR: ↑ p-AMPK ↑ GRP78, CHOP expression ↓ ROS production High-dose CUR: ↓ p-AMPK ↓ GRP78, CHOP expression ↑ ROS production	[219]
	MM-B1, H-Meso-1, MM-F1 and murine #40a malignant mesothelioma cells (25 µM)		Induction of autophagy, but autophagic flux blocked ↑ p62/SQSMT1, LC3 I	[220]
	TUBO murine Her2/neu^+^ breast cancer cells (25 μM + CQ)	Immunocompetent or immunocompromised BALB/c mice s.c. injected with TUBO cells (100 mg/kg, p.o., 3 times a week + CQ)	↓ p62 expression ↑ LC3 II expression Immunocompetent mice ↑ Tumor growth ↑ Autophagosomes in tumor tissues ↓ p62 expression ↓ Foxp3^+^ T regulatory cells ↑ CD8^+^ T cells Immunocompromised mice ↓ Tumor growth	[221]
	CAL-27, SCC-15 and FaDu HNSCC cancer cells (25 μM + Resveratrol)		Induction of autophagy ↑ LC3 I to LC3 II conversion ↑ LC3 II expression ↑ Autophagosomes	[222]
	SUP-B15 Ph^+^ acute lymphoblastic leukemia cells (30 µM)		Induction of autophagy ↑ RAF/MEK/ERK pathway ↑ Autophagy	[223]
	A172 glioblastoma cells (10 µM)		Induction of autophagy ↑ LC3 II expression ↑ LC3-puncta structures ↑ ATG5, ATG12, Beclin 1 expression	[224]
	Caki, ACHN and A498 renal carcinoma cells; U87MG glioma cells; MDA-MB-231 breast carcinoma cells (20 µM + PP242)		Induction of autophagy ↓ mTORC2/Akt pathway ↓ Rictor, Akt ↑ Cytosolic Ca^2+^, MMP, cytosolic pH ↑ Autophagy, Lysophagy, galectin-3 Colocalization of LAMP-1 and p62 ↑ LC3-puncta structures ↑ LC3 II expression ↑ p62 expression	[225]
	HCT-116, HT-29, HepG2 and Huh7 gastrointestinal cancer cells (2 µM + Sildenafil)		Induction of autophagy ↓ mTORC1 and mTORC2 activity ↑ Beclin 1 ↑ Autophagosome and autolysosome	[226]
CUR or solid lipid CUR particles (SLCP)	U87MG; mouse, GL261; rat, F98 glioblastoma cells; C6-glioma rat glial tumor cells; N2a cells mouse neuroblastoma cells (25 µM)		Induction of autophagy ↑ ATG5, ATG7, Beclin 1 ↑ LC3A/B-II/LC3A/B-I ratio ↓ Mitophagy markers PINK-1, NIP3L/NIX, BNIP3, HIH-1α ↓ Akt/mTOR pathway ↓ Akt, p-Akt, mTOR and p-mTOR ↓ LAMP-2a ↑ Autophagic vacuoles and membrane blebbing	[227]
CUR-LDH	MDA-MB-231 breast cancer cells (25–100 µg/mL + Photodynamic therapy)		↑ Autophagosomes	[228]
Curcumin DMC BDMC	SAS oral cancer cell line (CUR: 30 µM) (DMC, BDMC: 15 µM)		Induction of autophagy ↑ Autophagic vacuoles ↑ MDC-labelled vacuoles ↓ p-mTOR, ↑ AMPKα1, Vps34, ULK1, ATG16L1, ATG5 ↑ LC3, Beclin 1	[229]
Curcumin DMC BDMC TetrahydroCUR	SAS oral cancer cell line + Gefitinib (CUR: 20 µM) (DMC, BDMC: 5 µM)	BALB/c athymic nude mice s.c. injected with SAS cells (30 mg/kg, i.p., every two days + gefitinib)	Induction of autophagy ↑ Cleaved-caspase-3 ↓ MMP ↑ Autophagic vacuoles ↑ ATG5, p62/SQSTM1, ULK1, Vps34 ↑ LC3, Beclin 1 in vivo ↑ Beclin 1	[230]
	Chemotherapy-resistant HL60 human leukemia cells		Induction of autophagy ↑ LC3 II expression ↑ p62 expression	[231]
TetrahydroCUR	A549 NSCLC cells (10–130 µM)		Induction of autophagy ↑ AVOs ↑ Beclin 1 expression ↓ mTOR, p-mTOR, p-Akt ↓ p62 expression ↑ LC3 II/I ratio ↑ PI3K	[232]
CA-5f	A549, H1299 and H157 NSCLC cells; HUVEC umbilical vein endothelial cells; HepG2 HCC cells; HeLa cervical cancer cells; HEK293 embryonic kidney 293 cells (1–40 µM)	BALB/c nude mice s.c. injected with A549 cells (40 mg/kg, i.v., every two days)	Inhibition of autophagy ↑ SQSTM1 expression ↓ Autophagophores degradation Modulation of cytoskeleton protein, membrane trafficking, vesicles mediated transport in vivo ↑ LC3 II expression ↑ SQSTM1	[233]
ZYX01	A549 NSCLC cells		Induction of autophagy ↑ AMPK/ULK1/Beclin 1 pathway ↑ LC3 II/I ratio ↑ Beclin 1 ↓ p62 expression	[234]
MOMI-1	A549 NSCLC cells; MCF-7 breast cancer cells; HepG2 HCC cells (20 µM)		Induction of autophagy ↑ Autophagic vacuoles ↑ MDC-labelled vacuoles ↑ LC3-puncta structures ↓ p62 expression ↑ Beclin 1 ↑ LC3 I to LC3 II conversion	[235]
MTH-3	MDA-MB-231 breast cancer cells (10 µM)		Induction of autophagy ↑ LC3 expression ↑ p62 expression ↑ ATG5, ATG7, ATG12, Beclin 1	[236]
WZ35	HCCLM3 HCC cells (20 µg/mL)		Induction of autophagy ↑ Autophagic vacuoles Deregulated YAP signaling ↓ LC3 I/II ratio ↓ ATG7, Beclin 1 expression ↑ p62 expression	[237]
***Phenolic Acids***				
Ellagic acid	HOP62 and H1975 lung cancer cells (10–50 µM)	BALB/c nude mice s.c. injected with HOP62 cells (40 mg/kg, i.p., every 2 days)	Induction of autophagy ↑ LC3-positive autophagosomes ↑ LC3 II and ATG5 expression ↓ p62 expression ↑ Activation AMPK ↓ mTORC1 and Akt	[238]
	SKOV-3 ovarian cancer cells (36.6 µM)		Induction of autophagy ↑ LC3 II/I ratio ↑ Beclin 1 and ATG5 expression ↓ p62 expression ↑ Activation AMPK ↓ mTORC1 and Akt	[239]
Punicalagin	U87MG glioma cells (1–30 µg/mL)		Induction of autophagy ↑ LC3 II expression ↓ LC3 I expression ↑ LC3-puncta structures ↑ Activation AMPK and p27	[240]
Grias Nuberthii extract	RKO and SW613-B3 colon cancer cells (20, 30, 50 µg/mL)		Induction of autophagy ↑ Beclin 1 and LC3 II expression ↓ p62 expression	[241]
Gallic acid	Cal33 OSCC cells (0.1, 0.5, 1 mg/mL)		Induction of cytoprotective autophagy, but autophagic flux blocked ↑ LC3 I to LC3 II conversion ↑ Beclin 1 and ATG5-ATG12 expression ↑ p62 expression ↓ Autolysosome formation	[242]
Paeonol	A2780 and SKOV-3 ovarian cancer cells (0.6–1.2 mM)	BALB/c nude mice (nu/nu) s.c injected with A2780 cells (40 mg/kg, i.p., every 2 days)	Induction of cytoprotective autophagy ↑ LC3 I to LC3 II conversion ↑ Autophagosomes ↓ p62 expression ↓ Akt/mTOR pathway	[243]
PGG	DU145, PC-3, TRAMP-C2 prostate cancer cells (25–75 µM)		Induction of cytoprotective autophagy ↑ Autophagosomes ↑ LC3 II expression ↓ S6K and 4EBP1 ↑ Akt activation	[244]
Corilagin	SGC-7901 and BGC-823 gastric cancer cells (10–30 µM)		Induction of cytoprotective autophagy ↑ LC3 II expression ↑ Autophagosomes	[245]
CAPE + EECP	MDA-MB-231 breast cancer cells (CAPE: 25 µg/mL + EECP: 25–100 µg/mL)		Induction of autophagy ↑ LC3 II expression ↓ p62 expression	[246]
CAPE	C6 glioma cells (10 µM)		Induction of cytoprotective autophagy ↑ LC3 II/I ratio ↑ Autophagosomes ↑ AMPK activation	[247]
Decyl caffeic acid	HCT-116 colorectal cancer cells (40 µM)		Induction of cytoprotective autophagy ↑ ATG3, ATG16, Beclin 1 and LC3 I/II expression	[248]
Artepillin C	CWR22Rv1 prostate cancer (50–100 µM)		Induction of cytoprotective autophagy ↑ LC3 II expression	[249]
*Cinnamomum cassia* extracts	SASVO3 oral cancer cells (50–100 µg/mL)		Induction of cytoprotective autophagy ↑ AVOs ↑ LC3 I, LC3 II, ATG14, rubicon and p62 expression ↓ PI3K/Akt/mTOR pathway	[250]
Ferulic acid	HeLa and Caski cervical cancer cells (2.0–4.0 mM)		Inhibition of autophagy ↓ LC3 II, Beclin 1, ATG5-ATG12 expression	[251]
Tributyltin (IV) ferulate	HCT-116, HT-29, Caco-2 colon cancer cells (400 nM)		Induction of autophagy ↑ MDC-labelled vacuoles ↑ LC3 II and p62 expression	[252]
*p*-coumaric acid	N2a neuroblastoma cells (150–200 µM)		Induction of autophagy ↑ Autophagosomes ↑ LC3 II expression	[253]
*Ganoderma lucidum* methanolic extract	AGS gastric cancer cells (66.6 and 133.2 µM)		Induction of autophagy ↑ Autophagosomes ↑ LC3 II expression ↓ p62 expression	[254]
***Lignans***				
Honokiol	B16-F10, SKMEL-28 melanoma cancer cells (30–40 µM)		Induction of autophagy Formation of autophagosomes ↑ LC3 II expression and cytoplasmic accumulation ↓ Akt/mTOR pathway and Notch signaling	[255,256]
	MG-63 osteosarcoma cells (10–20 µg/mL)		Induction of autophagy ↑ LC3 II expression ↓ Akt/mTOR pathway	[257]
	ARO, WRO, SW579 thyroid cancer cells (20–60 µM)	BALB/cAnN.Cg-Foxn1nu/CrlNarl nude mice s.c. injected with ARO cells (5 or 15 mg/kg, p.o., every 3 days)	Induction of autophagy ↑ LC3 II expression ↓ p62 expression	[258]
	Neuro-2a and NB41A3 neuroblastoma cells (50 µM)		Induction of autophagy ↑ AVOs ↑ LC3 II/I ratio	[259]
	Drug sensitive (U87MG, murine GL261) and resistant (U87-MR-R9) glioma cells (40 µM + TMZ)		Induction of autophagy ↑ Autophagic cells percentage	[260]
	PC-3, LNCaP, murine Myc-CaP prostate cancer cells (40 µM)		Induction of cytoprotective autophagy ↑ LC3 II expression Formation of autophagic vacuoles ↑ LC3-puncta structures	[261]
	OC2 and OCSL OSCC cells (20–40 µM)	BALB/cAnN.Cg-Foxn1nu/CrlNarl nude mice s.c. injected with SAS cells (5 and 15 mg/kg, p.o., twice a week)	Induction of autophagy ↑ LC3 II expression	[262]
Honokiol + Magnolol	U87MG and LN229 glioma cells (40 µM, each)		Induction of cytoprotective autophagy ↑ LC3 II expression	[263]
Vitexin 6	T-47D breast cancer cells and RKO colon cancer cells (5–20 µM)		Induction of autophagy Formation of autophagosomes ↑ LC3 II conversion ↑ LC3 II and Beclin 1 expression	[264]
Licarin A	A549 and NCI-H23 NSCLC cells (10–25 µM)		Induction of autophagy ↑ AVOs ↑ Beclin 1, LC3 II mRNA levels ↓ p62 levels	[265]
Trachelogenin	HCT-116 colon cancer cells (5–10 µM)		Induction of autophagy ↑ AVOs ↑ Beclin 1 ↑ LC3 I to LC3 II conversion	[266]
Magnolin	HCT-116 and SW480 colon cancer cells (10–40 µM)	BALB/c athymic nude mice s.c. injected with HCT-116 cells (20 mg/kg, i.p., daily)	Induction of autophagy ↑ LC3 II expression ↓ p62 expression Accumulation of double membrane vesicles ↑ LC3-puncta structures	[267]
Justicidin A	HT-29 colon cancer cells (0.5–1.5 µM)	NOD-SCID mice s.c. injected with HT-29 cells (6.2 mg, p.o., daily)	Induction of autophagy ↑ LC3 I to LC3 II conversion ↑ LC3 II expression ↑ AVOs ↑ LC3-puncta structures ↓ p62 expression ↓ p-mTOR, p-p70S6K expression ↑ Beclin 1, ATG5-ATG12 expression	[268]
Pinoresinol	SKOV-3 ovarian cancer cells (10–40 µM)	Mice s.c. injected with SKOV-3 cells (40 mg/kg, i.p., thrice a week)	Induction of autophagy Formation of autophagic vesicles ↑ LC3 II expression ↑ Beclin 1 expression ↓ p62 expression	[269]
Sesamin	HeLa cervical cancer cells (50 µM)		Induction of autophagy ↑ Autophagosomes ↑ LC3 II expression ↑ Beclin 1 expression	[270]
	HT-29 and LS180 colon cancer cells (50 µM)		Induction of autophagy Formation of double membrane vacuoles ↑ LC3-puncta structures ↑ LC3 I to LC3 II conversion ↑ MDC-labelled vacuoles	[271]
Magnolol	H460, A549 and NCI-H1299 NSCLC cells (A549: 80 µM) (A549 and NCI-H1299: 10–20 µM)		Induction of autophagy Formation of autophagosomes ↑ MDC-labeled vacuoles ↑ ATG5, ATG12 expression ↑ LC3 II/I ratio ↓ p62 expression ↓ Akt/mTOR pathway	[272,273]
	SGC-7901 human gastric adenocarcinoma cells (40–80 µM)		Induction of autophagy ↑ AVOs	[274]
Arctigenin	MCF-7 breast cancer cells (1–200 µM)		Induction of autophagy ↑ LC3 II expression ↑ LC3 II/I ratio ↓ mTOR pathway	[275]
	R-SW480, R-SW620 cisplatin resistant colorectal cancer (100 µM + Cisplatin)		Induction of autophagy ↑ LC3 II expression ↑ p65 expression ↓ LC3 I expression	[276]
	HepG2 HCC cells (1.25–10 µM)		Inhibition of autophagy ↑ LC3 II expression ↑ Beclin 1 phosphorylation ↑ p62 levels	[277]
DFS	DU145 prostate cancer cells and SW480 colon cancer cells (10 µM)		Induction of cytoprotective autophagy ↑ LC3 II levels ↑ Autophagosomes and autolysosomes	[278]
***Stilbenes***				
Resveratrol	HL-60 AML cells (12.5–100 μM)		Induction of autophagy ↑ LC3 II, ATG5 and Beclin 1 expression ↑ LKB1/AMPK activation ↓ PI3K/Akt/mTOR pathway	[279]
	K562 CML cells (50 μM)		Induction of autophagy ↑ AMPK activation ↓ mTOR pathway ↑ LC3 II and ATG3 expression ↑ p62 expression	[280]
	HT-29 and COLO201 colon cancer cells (HT-29: 150 μM) (COLO201: 75 μM)		Induction of autophagy ↑ LC3 II expression ↑ Autophagic vacuoles ↑ LC3-puncta structures	[281]
	HK-2 and Ketr-3 renal carcinoma cells (12.5–100 μM)		Induction of autophagy ↑AMPK activation ↓ mTOR phosphorylation ↑ LC3, ATG5, ATG7 expression	[282]
	MHCC-97 HCC cells (20–100 μM)		Induction of autophagy ↑ LC3 II/I ratio ↑ Beclin 1 expression ↓ p62 expression ↑ LC3-puncta structures ↓ p-Akt/Akt ratio	[283]
	C33A, CaLo, and HeLa cervical cancer cells (30–50 μM)		Induction of autophagy ↑ Lysosomal permeability ↑ Lysosomal swelling and degranulation ↑ Vacuoles and autophagosomes	[284]
	CAR cisplatin-resistant oral carcinoma cells (50 μM)		Induction of autophagy ↑ AVOs ↑ MDC-labeled vacuoles ↑ LC3-puncta structures ↑ AMPK activation ↓ Akt activation ↑ ATG5, ATG7, ATG12, ATG14, ATG16L1, Beclin 1 and LC3 II expression	[285]
	U373 glioma cells (100 μM)		Induction of autophagy ↑ LC3-labeled vesicles	[286]
	U87 glioma cells (30 μM)		Induction of cytoprotective autophagy ↑ Autophagosomes ↑ ATG5, Beclin 1 and LC3 II expression ↓ Akt and p70S6K activation	[287]
	U251 glioma cells (150 μM)		Induction of cytoprotective autophagy ↑ MDC-labeled vacuoles ↑ LC3-puncta structures ↑ LC3 II and Beclin 1 expression	[288]
	OVCAR-3 and CAOV-3 ovarian cancer cells (30μM)		Induction of autophagy ↑ LC3 II and ATG5 expression	[289]
	SKOV-3 ovarian cancer cells (25 μM)		Induction of cytoprotective autophagy ↑ LC3 II and Beclin 1 expression ↑ MDC-labeled vacuoles	[290]
	OVCAR-3 and CAOV-3 ovarian cancer cells (120 μM)		Induction of autophagy ↑ LC3 II and Beclin 1 expression ↑ LC3-labeled vesicles ↑ Autophagosomes ↓ STAT3 activation	[291]
		Female nu/nu mice injected i.p. with GFP-labeled A2780 ovarian carcinoma cells (160 mg/kg, i.p., daily)	Induction of autophagy ↑ Autophagosomes	[292]
	Ishikawa endometrial carcinoma cells (20 μM)		Induction of cytoprotective autophagy ↑ LC3 II expression ↑ Autophagosomes ↑ p-ERK and p-AMPKα	[293]
	A549 NSCLC cells (50 μM)		Induction of autophagy ↑ p62 degradation ↑ LC3 II expression ↑ MDC-labeled vacuoles	[294]
	A549 and H1299 NSCLC cells (200 μM)		Induction of cytoprotective autophagy ↑ Beclin 1 expression ↑ LC3 II/I ratio ↓ p62 expression ↑ SIRT 1 expression ↓ Akt/mTOR pathway ↑ p38 MAPK activation	[295]
	PC9 NSCLC cells (40 μΜ + Gefitinib)		Induction of cytoprotective autophagy ↑ LC3B II expression ↑ MDC-labelled vacuoles	[296]
	A549 NSCLC cells (2.5 μM + Cisplatin)		Induction of autophagy ↑ Autophagosomes ↑ LC3 II expression ↓ p62 expression ↓ p-Akt ↑ LC3-puncta structures ↑ Autophagosomes	[297]
	MSTO-211H and H-2452 malignant mesothelioma cells (30 μM + Cisplatin)		Induction of cytoprotective autophagy ↑ LC3 A and Beclin 1 expression	[298]
	EC109 and EC9706 squamous esophageal carcinoma cells (10–150 μM)		Induction of cytoprotective autophagy ↑ MDC-labelled vacuoles ↑ AVOs ↑ LC3 II, ATG5 and Beclin 1 expression ↑ Autophagosomes	[299]
	B16 melanoma cells (25–100 μM)		Induction of cytoprotective autophagy ↑ LC3 II and Beclin 1 expression ↓ Akt/mTOR pathway	[300]
	MCF-7 breast cancer cells (64 μM)		Induction of autophagy ↑ LC3-puncta structure ↑ LC3 II expression ATG7, Beclin 1 and Vps34 expression unchanged ↓ Akt/mTOR pathway	[301]
	MCF-7 and MDA-MB-231 breast cancer cells (100 μM + Rapamycin)		Inhibition of rapamycin-induced autophagy ↓ LC3 II expression ↑ p62 expression ↓ Akt activation	[302]
	MCF-7 and MDA-MB-231 breast cancer cells (MCF-7: 84.6 μM + DOX) (MDA-MB-231: 108 μM + DOX)		Inhibition of DOX-induced autophagy ↓ LC3B expression ↓ Beclin 1 expression ↓ Akt activation	[303]
	MDA-MB-231 breast cancer cells (72 μM + Salinomycin)		Inhibition of salinomycin-induced autophagy ↓ LC3 expression ↓ Beclin 1 expression	[304]
	A431 epidermoid carcinoma cells (50 μM)		No autolysosome formation ↑ LAMP-2 expression ↑ LC3 II expression ↓ Rictor expression	[305]
Pterostilbene	HL-60 AML cells		Induction of autophagy ↑ LC3 II expression ↑ LC3-puncta structures Accumulation autophagic vacuoles Reduction in autophagic degradation	[306]
	Bcap-37 and MCF-7 breast cancer cells (50 μM)		Induction of cytoprotective autophagy ↑ LC3B II expression ↑ Autophagosomes	[307]
	CAR cisplatin-resistant human oral cancer cells (50 and 75 μM)		Induction of autophagy ↑ AVOs ↑ MDC-labelled vacuoles ↑ ATG5, ATG7, ATG12, Beclin 1 and LC3 II expression ↓ p-Akt	[308]
***Xanthones***				
α-Mangostin	GBM8401 and DBTRG-05MG glioblastoma cells (2.5–10 µM)	BALB/cA-*ν* (*ν*/*ν*) nude mice s.c. injected with GBM8401 cells (2 mg/kg, i.p., daily)	Induction of autophagy ↑ AVOs ↑ MDC-labelled vacuoles ↑ Autophagosomes ↑ LC3-puncta structures ↑ AMPK pathway ↓ mTORC1 activity	[309]
		ICR mice treated topically with DMBA/TPA (5 and 20 mg/kg, i.p., daily)	Induction of autophagy ↑ LC3, LC3 II, Beclin 1 expression ↓ LC3 I and p62 expression ↓ PI3K/Akt/mTOR pathway	[310]
	K562, KBM5 and KBM5-T135I CML cells (5–20 µM)		Induction of cytoprotective autophagy ↑ LC3 II expression ↑ Autophagic vacuoles	[311]
Gambogic acid	K562 CML cells (0.5–2 µM)		Induction of autophagy ↑ Autophagic vacuoles ↑ LC3 II and Beclin 1 expression ↓ p62 expression	[312]
	NCI-H441 NSCLC cells		Induction of autophagy ↑ Beclin 1 expression ↑ LC3 I to LC3 II conversion ↑ Autophagosomes	[313]
	TE13 esophageal cancer cells (0.25–1 µM)		Induction of autophagy, but autophagic flux blocked ↑ LC3 II expression ↑ Autophagosomes ↓ Akt/mTOR pathway	[314]
	HCT-116 and SW260 colon cancer cells (0.25–1.5 µM)	BALB/c mice s.c. injected with C26 cells (8 mg/kg, i.p., daily)	Induction of cytoprotective autophagy ↑ Autophagic vacuoles ↑ AVOs ↑ LC3 I to LC3 II conversion ↑ Beclin 1, ATG7, ATG5-ATG12 expression ↓ p62 expression ↓ Akt/mTOR pathway	[315]
Gambogenic acid	H1975, H460 lung cancer cells (1.25–25 µM)		Induction of autophagy ↑ Autophagosomes ↑ LC3-puncta structures ↑ LC3 II expression ↑ GSK3β activation ↓ Akt/mTOR pathway	[316]
Isogambogenic acid	A549 NSCLC cells and HeLa cervical cancer cells (1.5–12 µM)	BALB/cA nude mice s.c. injected with A549 cells (16 mg/kg, i.v., twice a week)	Induction of autophagy, but autophagic flux blocked ↑ Autophagic vacuoles ↑ LC3 II and Beclin 1 expression ↓ p70S6K phosphorylation Inhibition degradation p62 Inhibition acidification of vacuoles	[317]
Gartanin	Hep3B, HepG2, Huh7 HCC cells (10–40 µM)		Induction of cytoprotective autophagy ↑ AVOs ↑ LC3 I to LC3 II conversion ↑ LC3-puncta structures ↑ Autophagosomes and autolysosomes ↓ p62 expression	[318]
	T24 and RT4 urinary bladder cancer cells (10–25 µM)		Induction of autophagy ↑ LC3-puncta structures ↑ Autophagosomes ↑ LC3 I to LC3 II conversion ↓ p70S6K and 4E-BP1	[319]
	T98G glioma cells (10 µM)		Induction of autophagy ↑ LC3-puncta structures ↑ LC3 II and Beclin 1 expression ↓ p62 expression ↓ PI3K/Akt/mTOR pathway	[320]
	22Rv1 and PC-3 prostate cancer cells (6–24 µM)		Induction of autophagy ↑ LC3-puncta structures ↑ LC3 II expression	[321]
Formoxanthone C	MDR A549RT-eto NSCLC cells (20 µg/mL)		Induction of autophagy ↑ Autophagic vacuoles ↑ LC3-puncta structures ↑ LC3 I to LC3 II conversion ↑ Beclin 1 expression ↓ p-mTOR levels ↑ Sensitivity to etoposide	[322]
Mangiferin	Gemcitabine-resistant Mia-PaCa2 pancreatic carcinoma cells (5–20 µM)		Induction of autophagy ↑ LC3 II and Beclin 1 expression	[323]
*Gentiana dinarica* extract and norswertianin	U251 glioblastoma cells (Extract: 50 µg/mL; noswertianin 40 µM)		Induction of autophagy ↑ AVOs ↑ LC3 I to LC3 II conversion ↓ p62 expression ↓ Akt/mTOR pathway	[324]
Xanthone V_1_	HeLa cervical cancer cells (10–20 µM)		Induction of autophagy ↑ LC3-puncta structures ↑ LC3 I to LC3 II conversion ↓ p62 expression	[325]
Cudraxanthone D	Ca9-22 and SCC25 OSCC cells (50 µM)		Inhibition of autophagy ↓ Autophagic vacuoles	[326]

↑, increase/upregulation; ↓, decrease/downregulation. Abbreviations: 5-FU, 5-fluorouracil; 6-CEPN, 6-C-(E-phenylethenyl) naringenin; 8-CEPQ, 8-C-(E-phenylethenyl) quercetin; ALT, alanine aminotransferase; AML, acute myeloid leukemia; AMPK, AMP-activated protein kinase; AOM, azoxymethane; AST, aspartate aminotransferase; ATG, autophagy-related protein; AVO, acidic vesicular organelle; BDMC, bisdemethoxycurcumin; BNIP3L, BCL2/adenovirus E1B 19 kDa protein-interacting protein 3-like; C3G, cyanindin-3-*O*-glucoside; CA-5f, (3E,5E)-3-(3,4dimethoxybenzylidene)-5-[(1H-indol-3-yl)methylene]-1-methylpiperidin-4-one; CAPE, caffeic acid phenethyl ester; CHNQ, 3,7-dihydroxy-2-[4-(2-chloro-1,4-naphthoquinone-3-yloxy)-3-hydroxyphenyl]- 5-hydroxychromen-4-one; CHOP, DNA damage-inducible transcript 3 protein; CML, chronic myeloid leukemia; CQ, chloroquine; CUR, curcumin; DDP, cisplatin; DFS, [(−)-(2*R*, 3*R*)-1,4-*O*-diferuloylsecoisolariciresinol]; DMBA, 9,10-dimethylbenz[a]anthracene; DMC, demethoxycurcumin; DOX, doxorubicin; DSS, dextran sulfate; ECG, (−)-epicatechin-3-*O*-gallate; EECP, ethanol extract of Chinese propolis; EGCG, (−)-epigallocatechin-3-*O*-gallate; EGR1, early growth response protein 1; ERK, extracellular signal-regulated kinase; ETO, etoposide; FOXO3a, forkhead box protein O3; FUNDC1, FUN14 domain-containing protein 1; GQ, 7-*O*-geranylquercetin; GRP78, endoplasmic reticulum chaperone BiP; GTP, guanosine triphosphate; HCC, hepatocellular carcinoma; HDAC1, histone deacetylase 1; HLP, *Hibiscus sabdariffa* leaf polyphenolic; HNSCC, head and neck squamous cell carcinoma; HO-1, heme oxygenase 1; i.p., intraperitoneally; i.v., intravenously; I3C, indol-3-carbinol; JLP, c-Jun NH2-terminal kinase (JNK)-associated leucine zipper protein; JP8, 4-(S)- (2,4,6-trimethylthiobenzyl)- EGCG; LAMP, Lysosomal-Associated Membrane Protein; LC3, microtubule-associated protein 1A/1B-light chain 3; LDH, layered double hydroxide nanocomposite; LKB1, serine/threonine liver kinase B1(STK11); MDC, monodansylcadaverine; MMP, mitochondrial membrane potential; MPE, polyphenols of Mulberry water extract; MTH-3, Bis(hydroxymethyl) alkanoate curcuminoid derivative; mTOR, mammalian target of rapamycin; NOD/SCID, Non-Obese Diabetic/severe combined immunodeficiency disease; Nrf2, nuclear factor erythroid 2-related factor 2; NSCLC, non-small-cell lung cancer; OSCC, oral squamous cell carcinoma; p-, phospho; p.o., per os; PEF, low strength pulsed electric field; PGG, penta-*O*-galloyl-β-D-glucose; Ph^+^, Philadelphia chromosome-positive; PINK1, serine/threonine-protein kinase PINK1, mitochondrial; PP242, mTOR inhibitor; RCC, renal cell carcinoma; ROS, reactive oxygen species; s.c., subcutaneously; SEPW1, selenoprotein W; SQSTM1, Sequestosome-1; STAT, signal transducer and activator of transcription; TMZ, temozolomide; TPA, 12-*O*-tetradecanoylphorbol-13-acetate; TRAIL, tumor necrosis factor-related apoptosis-inducing ligand; ULK, unc-51-like autophagy-activating kinase; US, low energy ultrasound; Vps, phosphatidylinositol 3-kinase catalytic subunit type 3 (PIK3C3/ Vps34).
